# The microenvironment of ocular surface in keratoconus: a systematic review

**DOI:** 10.1186/s40662-025-00454-0

**Published:** 2025-09-24

**Authors:** Sana Niazi, Farideh Doroodgar, Stephen Pflugfelder, Kia Bayat, Seyed-Farzad Mohammadi, Maedeh Mazloomi, Jorge L. Alió del Barrio, Majid Moshirfar, Jorge L. Alió

**Affiliations:** 1 Translational Ophthalmology Research Center, Tehran University of Medical Sciences, Tehran, Iran; 2 Department of Ophthalmology, Baylor College of Medicine, Houston, TX USA; 3 Ophthalmic Research Center, Research Institute for Ophthalmology and Vision Science, Shahid Beheshti University of Medical Sciences, Tehran, Iran; 4 Cornea, Cataract and Refractive Surgery Unit, Vissum (Miranza Group), Calle Cabanal, 1, 03015 Alicante, Spain; 5 Division of Ophthalmology, School of Medicine, Universidad Miguel Hernández, Alicante, Spain; 6 John A. Moran Eye Center, University of Utah, Salt Lake City, Utah USA

**Keywords:** Keratoconus, Ocular surface, Dry eye disease, Cornea, Corneal epithelium

## Abstract

**Purpose:**

Keratoconus is a progressive corneal ectatic disorder characterized by thinning and irregularity of the cornea, significantly impairing visual acuity. Recent studies have explored how non-ectatic conditions, such as dry eye and tear film instability and alteration of the ocular surface microenvironment, contribute to the development and progression of keratoconus. This comprehensive review aims to investigate the complex relationship between keratoconus and ocular surface diseases by examining the microenvironmental changes that occur on the ocular surface throughout the course of keratoconus, as well as the related clinical implications.

**Methods:**

In this PROSPERO-registered study (ID: CRD42025643808), PubMed, Scopus, Cochrane, Embase, Web of Science, and Google Scholar were thoroughly searched to retrieve all pertinent papers published up to January 2025. The retrieved publications were then reviewed, and the eligible ones were included.

**Results:**

Keratoconus, with a similar inflammatory profile to that of ocular surface disease, has elevated Interleukin (IL)-1β, IL-6, tumor necrosis factor (TNF)-α, and matrix metalloproteinase (MMP)-9, contributing to extracellular matrix degradation and stromal thinning. Tear film instability, altered lipid secretion, and oxidative stress exacerbate disease progression. These findings suggest that keratoconus is not only a biomechanical disorder but also an inflammation-driven one.

**Conclusion:**

This study comprehensively reviews the intricate relationship between the ocular surface microenvironment and keratoconus. Managing this microenvironment in keratoconus patients, as well as inflammation, oxidative stress, and tear film dysfunction, can potentially improve patient outcomes.

**Supplementary Information:**

The online version contains supplementary material available at 10.1186/s40662-025-00454-0.

## Background

Keratoconus (KC), the most common form of corneal ectasia, is a bilateral corneal disease characterized by the progressive steepening and thinning of the cornea, resulting in irregular astigmatism and subsequently impaired vision [1]. The lowest and highest prevalence of KC were respectively reported in Russia (0.0004%) [2] and 6- to 21-year-old individuals in Saudi Arabia (4.79%) [3]. Additionally, according to the results of a German cohort study, KC was detected in 0.49% of a predominantly Caucasian population, indicating a tenfold higher rate than previously reported [4].

Recent clinical observations have revealed that many patients with KC exhibit typical symptoms of ocular surface diseases (OSDs), such as dry eye disease (DED) [5, 6]. Moreover, inflammatory mediators associated with DED, meibomian gland dysfunction (MGD) [7–9], and blepharitis [10], as well as eye rubbing due to atopic keratoconjunctivitis (AKC), could potentially contribute to the initiation or progression of KC [11–13]. Furthermore, specific inflammatory mediators may play a crucial role in the co-occurrence of KC and certain OSDs [14, 15]. These findings suggest that changes in the ocular surface microenvironment can play a critical role in KC development. Consequently, developing an integrated view regarding the intricate relationship between KC and the ocular surface’s integrity is paramount [16]. This review aims to (1) describe ocular surface microenvironmental changes observed in KC, (2) explore mechanisms by which KC may contribute to OSD pathogenesis, and (3) assess how KC therapies, particularly contact lenses and corneal cross-linking (CXL), impact the ocular surface in the context of coexisting OSD.

## Methods

### Search strategy

This study was registered in the International Prospective Register of Systematic Reviews (PROSPERO) with the following registration ID: CRD42025643808. To conduct this study, PubMed, Scopus, Cochrane, Embase, and Web of Science online databases were thoroughly searched to identify pertinent papers published between 1990 and May 2024. Both MeSH (medical subject heading) terms and non-MeSH terms were used. MeSH terms included “Ocular Surface Diseases,” “Keratoconus,” “Dry Eye Syndromes,” “Corneal Ectasia,” and “Inflammation Mediators,” and non-MeSH keywords such as “tear film instability,” “Meibomian gland dysfunction,” “pellucid marginal degeneration,” “keratoglobus,” “iatrogenic ectasia,” “contact lenses,” “collagen crosslinking,” “diagnostic challenges,” and “management strategies” were used to refine the search. Boolean operators (AND/OR) were applied to maximize sensitivity and specificity. In addition, a web-based search was carried out in Google Scholar using the terms ((“ocular surface”) OR (“meibomian gland dysfunction”) OR (“dry eye”) OR (“allergic conjunctivitis”)) AND ((keratoconus) OR (“pellucid marginal degeneration”) OR (“corneal ectasia”) OR (“iatrogenic ectasia”)).

Moreover, a follow-up search was conducted in the same Manner to include any new updates in the field by reviewing articles published between May 2024 and June 2025, and newer articles were added.

### Inclusion and exclusion criteria

Studies were included if they met the following criteria:

- Investigated the relationship between OSDs and KC, particularly in relation to disease progression, diagnosis, or treatment
- Provided original research data from clinical, observational, or interventional studies involving human subjects
- Examined the impact of OSDs (such as DED, MGD, blepharitis, or allergic conjunctivitis) on KC pathophysiology or management
- Assessed diagnostic challenges or treatment outcomes in KC patients with concurrent OSDs

Studies were excluded if they met any of the following criteria:

- Non-original research, including commentaries, expert opinions, narrative reviews, or letters to the editor
- Case reports or small case series that lacked sufficient sample sizes for broader applicability
- Retracted publications or studies with methodological limitations, comprising data integrity
- Ex vivo or animal studies
- Studies that did not meet predefined quality assessment standards, particularly those with a low AMSTAR score

### Study selection

I. Title and abstract screening: two independent reviewers conducted an initial assessment of all retrieved articles by reviewing their titles and abstracts to determine relevance. Articles that clearly fail to meet the inclusion criteria were excluded at this stage.
II. Full-text evaluation: The full texts of potentially eligible studies were then examined by the reviewers. Any disagreements about study eligibility were resolved through discussion between the two reviewers, with a third reviewer involved when necessary.
III. Quality evaluation: The methodological quality of the included studies was assessed using the quality assessment tool developed by the National Heart, Lung, and Blood Institute (NHLBI) for both observational cohort and cross-sectional studies, as well as systematic reviews (see Supplementary File 1). The evaluation considered three main criteria: (A) the validity of the study’s results, (B) the findings themselves, and (C) their relevance to local contexts. Studies were rated as “good,” “fair,” or “poor,” with only those rated “good” or “fair” being included in the final analysis.

### Data extraction

Two independent reviewers performed data extraction, with discrepancies resolved through discussion or consultation with a third reviewer. Extracted information included the first author and year, study design and quality rating, sample size (KC/control), outcomes assessed, biomarkers or parameters measured, main findings, and direction of change.

**Fig. 1:**
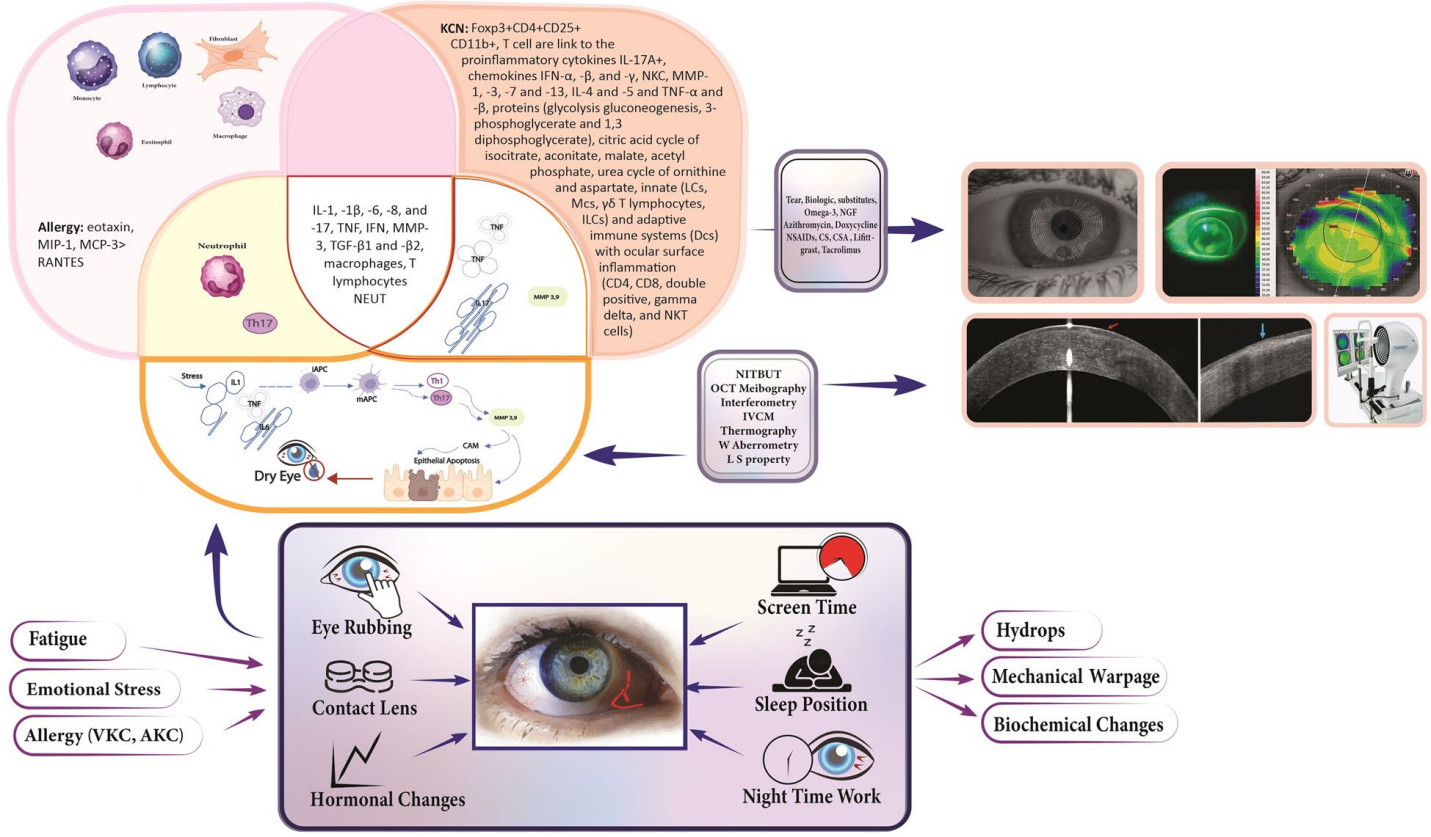
Schematic representation of the ocular surface microenvironment in keratoconus, summarizing the interplay between immunological mediators, environmental and behavioral factors, and comorbid conditions (©SN, FD, adapted with attribution from [253], CC BY 4.0 license). AKC, atopic keratoconjunctivitis; CD, cluster of differentiation; CD11b, cluster of differentiation11b; CS, corticosteroid; CsA, cyclosporine A; Dcs, dendritic cells; Foxp3, forkhead box P3; IFN, interferon; IL, interleukin; ILC, innate lymphoid cells; IVCM, in vivo confocal microscopy; KCN, keratoconus; LC, lamina cribrosa; L S property, limbal stem cell property; MC, mast cells; MCP, monocyte chemoattractant protein; MIP, major intrinsic polypeptide; MMP, matrix metalloproteinase; NEUT, neutrophils; NGF, nerve growth factor; NITBUT, noninvasive tear break-up time; NKC, natural killer cells; NSAID, nonsteroidal anti-inflammatory drugs; OCT, optical coherence tomography; RANTES, regulated on activation, normal T-cell expressed and secreted; TGF, transforming growth factor; TNF, tumor necrosis factor; VKC, vernal keratoconjunctivitis; W Aberrometry, wavefront aberrometry

Given the broad scope of this review, encompassing the molecular, biochemical, structural, and clinical domains of KC and its relationship to OSD, we anticipated significant heterogeneity across studies. Indeed, notable variability was observed in biomarker quantification methods (e.g., tear cytokines), outcome reporting formats [e.g., Interleukin (IL)-6 in pg/mL vs. fold-change], study populations, and comparator groups (Fig. 1).

### Data synthesis

Given the heterogeneity in study designs, populations, OSD subtypes, and outcome measures, a narrative synthesis was conducted. Studies were grouped and analyzed thematically based on the nature of the OSD and its relationship with KC, including aspects such as pathophysiology, diagnostic challenges, and treatment implications.

While meta-analysis is a powerful tool for data pooling, it requires consistent outcome definitions, standardized measurements, and comparable study designs. In this review, meta-analysis was only feasible for a limited subset of outcomes. However, restricting quantitative synthesis to such a narrow fraction would not adequately reflect the comprehensive scope of this review and could be misleading.

Therefore, we performed a structured narrative synthesis supported by summary tables outlining study characteristics, sample sizes, outcome directionality, and quality ratings.

## Results

Before initiating the screening process, all retrieved search results were imported into EndNote X9 (Clarivate, Philadelphia, PA, USA). Following the consolidation of search outputs, 5,831 records were identified. Duplicate entries were then removed, and a follow-up search contributed additional records.

**Fig. 2:**
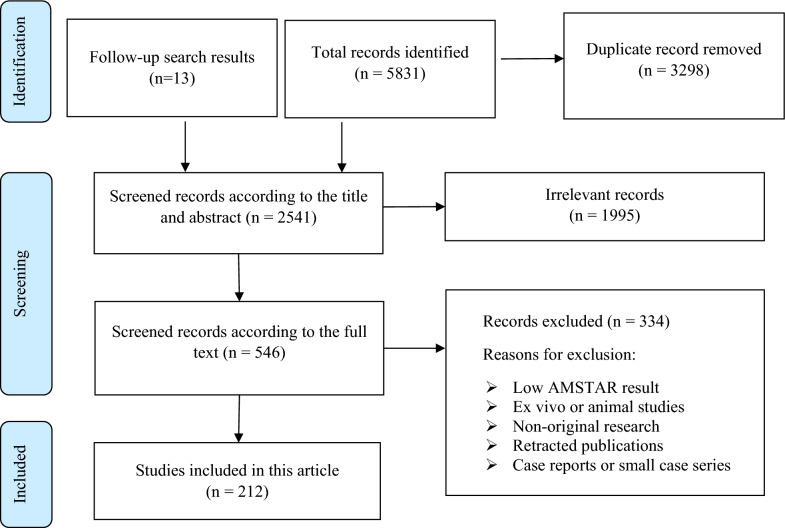
Preferred reporting items for systematic reviews and meta-analyses (PRISMA) flowchart. The key characteristics of all included studies are summarized in Table 1

In total, 212 studies were selected for inclusion in this review. The study selection process is detailed in the Preferred Reporting Items for Systematic reviews and Meta-Analyses (PRISMA) flowchart (Fig. 2).

**Table 1:** Characteristics of the included studies

First author	Study Design	Participants	Interventions/exposures & comparators	Outcomes	Study Quality
Flattau et al. 1991 [17]	Symposium proceedings chapter, expert consensus/review	Contact lens wearers with GPC	Various strategies (lens withdrawal, conjunctival irrigation with saline)	Symptom relief, improved lens tolerance	Fair
Wilson et al. 1996 [18]	Experimental (animal + in vitro)	Mouse corneas; in vitro stromal fibroblasts	Epithelial debridement vs. controls; IL‑1 exposure	Keratocyte apoptosis is linked to epithelial injury and IL‑1 signaling	Good
Irkec et al. 1999 [19]	Cross-sectional observational	Soft contact lens wearers with and without GPC	Comparison of tear LTC4 levels	Elevated LTC4 in GPC patients (*P* < 0.05), implicating an inflammatory mediator	Fair
Cheng et al. 2001 [20]	Case-control	KC vs. normal corneal buttons	Expression levels of type XII collagen, BP180, and integrins	Reduced type XII collagen in KC; other proteins unchanged	Good
Miyoshi et al. 2001 [21]	Cross-sectional clinical	Patients undergoing conjunctival brush cytology	IL‑8 levels correlated with cell infiltrates	IL‑8 correlates with neutrophil/eosinophil infiltration and corneal damage	Good
Saghizadeh et al. 2001 [22]	Comparative molecular analysis (RT-PCR)	Corneas with KC, bullous keratopathy, diabetic, and controls	Expression of IL‑1α, IL‑8, PDGF-B, BMP-2/4, IGF‑I, TGF‑β2, FGF‑2, VEGF	Altered cytokine/growth factor expressions in diseased corneas	Good
Sangwan 2001 [23]	Narrative review	Published literature on limbal stem cells, no new data	N/A	Role of limbal stem cells in corneal maintenance and disease	Fair
Schaumberg et al. 2001 [24]	Observational cohort	~ 25,000 postmenopausal women	HRT (estrogen alone or combined) vs. none	Estrogen alone: OR 1.69; combined: OR 1.29; each 3‑year HRT + 15% dry eye risk	Good
Erie et al. 2002 [25]	Observational	KC patients vs. controls	Keratocyte density via confocal microscopy	Reduced stromal keratocyte density in KC	Fair
Goto et al. 2002 [26]	Cross-sectional inical	Dry eye patients vs. healthy controls	Functional visual acuity testing under conditions	Impaired functional visual acuity in the dry eye group	Fair
Brookes et al. 2003 [27]	Observational histological	Corneal buttons from 10 patients with advanced KC vs. controls	Microscopy labeling of nerves, keratocytes (Cathepsin B/G, α-tubulin)	Corneal nerves thicken and express higher cathepsins in KC; nerves may facilitate degradation of Bowman’s layer and anterior stroma	Good
Dogru et al. 2003 [5]	Prospective case-control clinical	38 KC patients (75 eyes) vs. 40 controls (80 eyes)	Ocular exams including corneal sensitivity, Schirmer’s test, tear break-up time, fluorescein/rose bengal staining, and impression cytology	Reduced corneal sensitivity and tear function; increased staining, goblet cell loss, squamous metaplasia—all worsening with KC severity	Good
McCulley and Shine 2003 [28]	Narrative review	Synthesizes published research on meibomian gland physiology and tear lipid	N/A	Summarized the meibomian gland’s role in the tear lipid layer, tear stability, and evaporative dry eye	Fair
Wollensak et al. 2003 [29]	Non-randomized interventional pilot study	Progressive KC patients	Corneal collagen cross-linking (riboflavin + UVA) vs. untreated historical controls	Halted progression; increased corneal rigidity; reduced keratometry readings	Good
Bron and Tiffany 2004 [7]	Narrative review	Literature-based	N/A	Described the pathophysiology of meibomian gland disease and its contribution to evaporative dry eye	Fair
Bron et al. 2004 [30]	Experimental review	Evaluation of lipid layer mechanisms using existing data	N/A	Functional aspects: lipid secretion, spread, evaporation control, and tear stability	Fair
Ermis et al. 2004 [31]	Prospective interventional	N/A	Topical dexamethasone vs. ciprofloxacin eye drops; pre/post bacterial cultures	Dexamethasone increased conjunctival flora; ciprofloxacin decreased it	Fair
Georgiou et al. 2004 [32]	Retrospective cohort analysis	Asian vs. White patients with KC, incidence of atopy recorded	Ethnicity-based comparison	Asians had higher KC incidence and more atopic disease	Fair
Szczotka-Flynn 2004 [33]	Narrative review	Literature review on ocular surface factors affecting corneal topography	N/A	Discussed tear film, eyelid, contact lens, and ocular surface influences on topography readings	Fair
Takano et al. 2004 [34]	Cross‑sectional cytology-based	Atopic keratoconjunctivitis patients with corneal lesions	Brush cytology assessing inflammatory cell counts by lesion severity	Positive correlation between inflammatory cells and lesion severity	Good
Hollingsworth et al. 2005 [35]	Prospective observational	KC patients (mean age 31 ± 10 years) and a small histological sample	Slit-scanning confocal microscopy vs. light microscopy	Correlated epithelial abnormalities, Bowman’s layer breaks, stromal haze, and keratocyte changes with histopathology	Good
Lema and Duran 2005 [36]	Prospective case-control	28 KC patients and 20 healthy controls	Tear levels of IL‑4, IL‑6, IL‑10, TNF‑α, ICAM‑1, VCAM‑1, MMP‑9	Significantly higher IL‑6, TNF‑α, and MMP‑9 in KC tears; levels correlated with disease severity	Good
Mannion et al. 2005 [37]	Prospective comparative observational	13 KC subjects *vs.* 13 age-matched controls	Confocal microscopy & esthesiometry to assess nerve morphology and sensitivity	KC showed reduced nerve fiber density, increased stromal nerve thickness, altered nerve orientation; contact lens-wearing KC had decreased corneal sensitivity	Good
Nepp et al. 2005 [38]	Interventional clinical case series	Keratoconjunctivitis sicca patients	Topical cyclosporin A treatment	Improvement in DED symptoms	Fair
Stern et al. 2005 [39]	Observational review	Dry eye and allergic conjunctivitis patients	N/A	Evaluated ocular surface inflammation markers	Fair
Moon et al. 2006 [40]	Prospective clinical observational	KC patients, some with contact lens use	Ocular surface exams comparing contact lens wearers against non-wearers	Contact lens usage is associated with increased ocular surface changes—staining and tear film instability	Fair
Patel and McGhee 2006 [41]	Case series	Four KC eyes	Laser scanning confocal microscopy for wide-area sub‑basal nerve mapping	Abnormal nerve architecture: tortuous bundles, closed loops, lower nerve density centrally	Fair
Smith et al. 2006 [42]	Laboratory molecular analysis	KC vs. normal corneas	MMP‑2 activation and TIMP expression assays	Demonstrated increased MMP‑2 activation and altered TIMP levels in KC tissue	Good
Katsifis et al. 2007 [43]	Narrative review	Literature on T lymphocyte roles in Sjögren’s syndrome	N/A	Discussed T-cell contributions to Sjögren’s immunopathology	Fair
Mazzotta et al. 2007 [44]	Prospective observational	KC patients receiving CXL	Post-riboflavin-UVA cross-linking evaluation	Reported stromal haze after CXL via in vivo confocal microscopy	Good
Acera et al. 2008 [45]	Cross-sectional observational	77 patients with ocular surface diseases (blepharitis, allergy, dry eye, conjunctivochalasis) and 18 controls	Tear levels of IL‑1β, IL‑6, and pro‑MMP‑9 across groups	IL‑1β & IL‑6 elevated in conjunctivochalasis; pro‑MMP‑9 significantly higher in all disease groups vs. controls	Good
Bradley et al. 2008 [46]	Case-control serological assay	Six dry eye patients and six healthy controls	Serum levels of various growth factors (TGF‑β1/2, NGF, IGF‑1, EGF, FGF, KGF, HGF, VEGF, PDGF, BDNF, NT‑3, GDNF)	No significant differences between the dry eye and control groups	Fair
Eberwein, et al. 2008 [47]	Case report	Single KC patient undergoing deep lamellar keratoplasty post‑crosslinking	CXL followed by DALK	Reported corneal melting post-surgery	Bad
Mazzotta et al. 2008 [48]	Prospective observational	KC patients treated with CXL	Corneal healing tracked via in vivo confocal microscopy	Documented early keratocyte apoptosis, nerve regeneration, and late repopulation patterns	Good
Ebrahimi et al. 2009 [49]	Narrative review	Literature on limbal stem cells	N/A	Overview of limbal stem cell biology, isolation, transplantation, and disease relevance	Fair
Koller et al. 2009 [50]	Retrospective cohort	KC patients post‑CXL	Outcomes and complications tracked	Low failure rate (1.3% progression), some stromal haze, persistent epithelial defects, sterile infiltrates	Good
McMonnies 2009 [51]	Narrative review	Existing literature on corneal trauma	Eye rubbing mechanisms in KC	Discussed mechanical trauma, inflammatory mediators, and oxidative stress as rubbing-related risk factors in KC	Fair
Patel et al. 2009 [52]	Prospective observational	Moderate‑to‑severe KC patients and controls	In vivo confocal microscopy + aesthesiometry	Lower nerve density and corneal sensation in KC vs. controls	Good
Reinstein et al. 2009 [53]	Retrospective	KC suspects and normal eyes	Corneal epithelial thickness mapping via Artemis high-frequency ultrasound	Detected localized thinning in KC/blends, aiding detection of early KC	Good
Tomlinson et al. 2009 [54]	Narrative review	Literature on lacrimal system physiology	N/A	Summarized tear production, evaporative loss mechanisms, blink function, drainage—all essential for tear film balance	Fair
Fink et al. 2010 [55]	Prospective cohort	Men, pre-/post-menopausal women with KC	Corneal measures tracked across gender and hormone status	KC progressed in both genders and across hormone statuses; no significant differences observed	Good
Lema et al. 2010 [56]	Cross-sectional proteomic analysis	KC patients vs. controls	Tear protein profiling using sodium dodecyl sulfate–polyacrylamide gel electrophoresis (SDS-PAGE) and mass spectrometry	Identified 18 differentially expressed proteins, including lower SFRP1 and increased proteases	Good
Montes-Mico et al. 2010 [57]	Narrative review	Synopsis of tear film–ocular optical quality literature	Various optical test methods (double-pass, wavefront, videokeratoscopy)	Tear film stability is critical to optical quality; tear breakup increases ocular aberrations	Fair
Pannebaker et al. 2010 [58]	Cross-sectional	KC patients [gas permeable (GP) lens wearers and non-wearers] vs. controls	Tear protein analysis via LC–MS/MS	Revealed distinct protein profiles; supports the tear film as a KC biomarker source	Good
Stabuc-Silih et al. 2010 [59]	Narrative review	Review of genetic and clinical KC studies	Summarized linkage analyses, candidate gene evaluations	Discussed genetic heterogeneity and phenotype-genotype discrepancies in KC	Fair
Zheng et al. 2010 [60]	Experimental animal/in vitro	Mouse ocular tissues under desiccating stress (DS)	DS exposure vs. controls; evaluated dendritic cell and cytokine expression	DS enhances corneal/conjunctival production of IL‑6, TGF‑β1/2, IL‑23, IL‑1β, promoting Th17 differentiation	Good
Corrales et al. 2011 [61]	Cross-sectional clinical study	Dry eye patients & healthy controls	Conjunctival mucin gene expression (e.g., MUC1, MUC4) via impression cytology & qPCR	Decreased mucin gene expression in DES—potential diagnostic biomarkers	Fair
Jun et al. 2011 [62]	Cross-sectional	KC patients vs. controls	Multiplex bead assays and ELISA for IL‑4, IL‑6, TNF‑α, IL‑17, etc	IL‑6 increased; TNF‑α not elevated as earlier suggested; decreased IL‑4 and increased IL‑17 in KC tears	Good
Liu et al. 2011 [63]	Prospective experimental	40 healthy subjects	Eye rubbing episodes and breath-holding assessed via ocular response analyzer	Eye rubbing significantly lowered corneal hysteresis, corneal resistance factor, and Goldmann IOP—no change from breath holding	Good
Marchitti et al. 2011 [64]	Narrative review	Compiles cellular responses to UV stress and corneal ALDH enzymes	N/A	UV-induced oxidative stress mitigated by ALDH enzymes; relevance for corneal clarity and UV protection	Fair
McMonnies 2011 [65]	Commentary/brief report	N/A	Discussion of blink efficiency metrics	Proposed that blink completeness and efficiency are critical yet overlooked in ocular surface disease management	Fair
Sangwan et al. 2011 [66]	Retrospective case series	49 eyes of 27 VKC patients	Clinical and cytologic evaluation	LSCD in ~ 1.2% of > 2,200 VKC eyes; associated with longer disease duration and older age; clinical plus cytological evidence (goblet cells on cornea)	Good
Balasubramanian et al. 2012 [67]	Cross-sectional observational	KC patients vs. controls	Tear analysis of proteases, proteolytic activity, and inflammatory molecules	Elevated tear proteases and inflammatory markers in KC	Good
Kanellopoulos et al. 2012 [68]	Observational comparative	Normal, untreated ectatic, and post-CXL ectatic corneas	Epithelial thickness mapping with AS-OCT	Altered epithelial thickness in early ectasia; thicker profiles post‑CXL	Good
Kaur and Mutus 2012 [69]	Experimental molecular review	Literature-based on platelet and thymosin β4 interactions	N/A	Thymosin β4’s role in platelet function, wound healing, and cytoprotection	Fair
Matalia et al. 2012 [70]	Ex vivo experimental	Cultured human limbal epithelial cells	UVA‑riboflavin exposure vs. controls	UVA induced apoptosis in limbal epithelial cells—implications for CXL safety	Good
McMonnies et al. 2012 [71]	Narrative review	Theoretical discussion on the mechanical effects of heat in eye rubbing and massage	N/A	Proposed that heat from rubbing contributes to corneal deformation and disease progression	Fair
Pucker and Nichols 2012 [72]	Review	Literature summary of meibum and tear lipid analysis	N/A	Discussed lipid composition, meibum biophysics, and tear film stability	Fair
Balasubramanian et al. 2013 [73]	Experimental clinical	KC patients and healthy controls	Tear measures before/after eye rubbing	Eye rubbing increased tear protease levels, activity, and inflammatory cytokines—linking mechanical insult to KC pathogenesis	Good
Caporossi et al. 2013 [74]	Prospective clinical follow-up	Progressive KC patients undergoing transepithelial CXL	Transepithelial CXL for 24 months (no control group)	Improvement in visual acuity and stabilization of keratometry readings; minimal haze	Fair
Cho et al. 2013 [75]	Prospective case-control study	31 asymmetrical KC patients (31 KC eyes, 31 subclinical eyes) + 30 controls	Corneal sensitivity (Cochet–Bonnet), Schirmer’s test, tear osmolarity, and impression cytology	Reduced sensitivity, lower Schirmer score, increased squamous metaplasia and goblet loss in KC/subclinical eyes vs. controls; tear osmolarity unchanged	Good
Ghosh et al. 2013 [76]	Exploratory proteomic and gene expression case series	KC patients (sample size unclear) vs. controls	Tear and corneal tissue analysis via proteomics and RT-PCR	Identified differential protein expression and mRNA patterns associated with KC	Fair
Jeyalatha et al. 2013 [77]	Experimental interventional clinical	KC patients undergoing CXL	Use of PMMA ring to protect limbal stem cells during CXL	PMMA ring appeared to reduce epithelial damage to the limbus; no control group	Fair
Kontadakis et al. 2013 [78]	Prospective interventional case series	24 KC patients (30 eyes)	Standard epi-off CXL	Transient decrease in corneal nerve density and sensitivity for 6 months; tear secretion and film stability unaffected	Good
Ma 2013 [79]	Narrative review (NRF2 in oxidative stress)	Review of cellular/animal studies	N/A	Summarizes NRF2’s role in regulating oxidative responses and protection mechanisms	Fair
Rocha, et al. 2013 [80]	Cross-sectional imaging study	KC eyes, post-ectasia, and controls	Spectral-domain OCT epithelial thickness mapping	Epithelial thinning patterns distinct in KC and ectasia vs. controls—useful for screening in post-refractive cases	Good
Roy et al. 2013 [81]	Perspective commentary	N/A	Theoretical discussion of corneal biomechanics	Outlines mechanical stiffening loss in KC; advocates biomechanical evaluation	Fair
Spadea et al. 2013 [82]	Systematic literature review	Studies evaluating corneal sensitivity in KC	N/A	Consolidates findings—KC patients consistently show decreased sensitivity	Good
Taneri et al. 2013 [83]	Prospective clinical study	KC patients undergoing CXL	Evaluation of dry eye parameters [Schirmer, tear film break-up time (TFBUT), staining, symptom score] pre-/post-CXL	Significant transient deterioration in tear film metrics and increased symptoms at 1 month post-CXL, normalizing by 3 months	Good
Wojcik et al. 2013 [84]	Narrative review (oxidative stress in corneal disease)	Review of KC and Fuchs dystrophy pathogenesis	N/A	Highlights the role of ROS, antioxidant enzymes, and oxidative pathways in corneal dystrophies	Fair
Cui et al. 2014 [85]	Cross‑sectional observational	100 symptomatic dry-eye patients vs. 35 controls	Fourier-domain OCT to measure corneal epithelial thickness; dry eye severity assessed via OSDI, TBUT, Schirmer, and staining	Superior corneal epithelium thinner in dry eye (≈ 50.6 µm) vs. controls (≈ 52.0 µm; *P* = 0.037); thinning correlated with disease severity	Good
Gagliano et al. 2014 [86]	Case-control	22 postmenopausal women with severe evaporative dry eye vs. 22 without	Serum 17-β‑estradiol, estrone, testosterone vs. ocular tests (TBUT, Schirmer, osmolarity)	Lower sex hormone levels in cases (*P* < 0.05); inversely correlated with osmolarity (r = –0.7 to –0.88)	Good
Hawkes and Nanavaty 2014 [87]	Literature review	Summarizes studies on eye rubbing and KC	N/A	Concludes that repeated rubbing exerts mechanical and inflammatory effects contributing to KC progression	Fair
Hong et al. 2014 [88]	Cross-sectional observational	Long-term soft contact lens wearers (n unspecified) vs. non-wearers	OCT epithelial thickness maps, tear film assessments, and corneal staining	Contact lens wear associated with regional epithelial thinning, reduced tear stability, and increased staining	Fair
Kanellopoulos and Asimellis 2014 [89]	Pilot clinical	Subjects with dry eye and controls	3D OCT epithelial thickness mapping	Dry eye patients had increased epithelial thickness variability; a preliminary tool for dry eye detection	Fair
Moore et al. 2014 [90]	Laboratory ex vivo	Human corneas with intact epithelium	CXL with and without epithelial protection using devices	Limbal stem cells protected using shields showed reduced UVA damage, suggesting safety benefits	Good
Shaheen et al. 2014 [91]	Narrative review	Broad literature on corneal innervation	N/A	Summarizes nerve anatomy, pathology, and implications in sensory and trophic diseases	Fair
Stojanovic et al. 2014 [92]	Contralateral prospective study	20 KC patients, each eye randomized to epi-on or epi-off CXL	Hypotonic riboflavin CXL with vs. without epithelial removal	Both protocols halted KC progression with similar efficacy; epi-on had reduced pain and faster recovery	Good
Sundaresan et al. 2014 [93]	Observational genetic association	Indian patients with KC and primary open‑angle glaucoma	Analysis of collagen-related gene variants	Reported specific gene associations, suggesting shared pathophysiology	Fair
Arita et al. 2015 [94]	Prospective interventional	15 controls and 15 MGD patients	Eyelid warming device used daily for 2 weeks	Improved tear film lipid layer thickness, TBUT, meibum quality; symptoms reduced	Good
Cejka and Cejkova 2015 [95]	Narrative review	Literature on oxidative stress and corneal injury	N/A	Highlights how UV, pollution, and chemical injuries induce ROS-mediated corneal damage and opacity; discusses antioxidant treatments	Fair
Dienes et al. 2015 [96]	Prospective case-control	19 KC vs. 20 healthy controls	Mechanical, chemical, thermal evoked corneal sensitivity using gas esthesiometer; tear secretion; NIBUT	KC patients demonstrated significantly elevated thresholds across stimulus types (e.g., mechanical 139.2 vs. 109.1 ml/min; *P* < 0.001), reduced tear secretion, and higher OSDI scores—no correlation with severity	Good
Galvis et al. 2015 [97]	Narrative review	Review of clinical/laboratory findings in KC	N/A	Proposes KC has inflammatory components—elevated cytokines, MMPs, and immune cell activity—suggesting disease is not purely biomechanical or genetic	Fair
Gordon-Shaag et al. 2015 [98]	Case-control	Israeli patients with and without KC	Questionnaire-based exposures; ocular biometric factors	Identified eye rubbing, familial history, and allergy as significant risk factors; skin allergy had the strongest association (OR ≈ 3.8)	Good
Karamichos et al. 2015 [99]	Experimental prospective	KC and control patients	Tear metabolomics via mass spectrometry	KC tears showed alterations in metabolites (e.g., amino acids, lipids) related to oxidative stress and matrix remodeling	Good
McMonnies 2015 [15]	Commentary/narrative review	Literature synthesis	N/A	Reviews inflammatory pathways in KC—eye rubbing, cytokine changes, oxidative stress; emphasizes inflammation’s role	Fair
Naderan et al. 2015 [100]	Cross-sectional clinical study	N/A	Demographic and clinical risk factors assessed	Reported associations between family history, allergy, eye rubbing, and disease severity	Fair
Shetty et al. 2015 [101]	Prospective experimental clinical	N/A	Tear MMP‑9 and cytokine levels measured before/after topical cyclosporine A	Cyclosporine A treatment decreased MMP‑9 and cytokines in tears, suggesting an anti-inflammatory benefit in KC	Good
Sorkhabi et al. 2015 [102]	Cross-sectional observational	KC patients vs. controls	Tear collection and inflammatory cytokine analysis	Elevated inflammatory mediators (IL‑6, TNF‑α, etc.) in KC tears compared to controls	Good
Sykakis et al. 2015 [103]	Systematic review & meta‑analysis	Patients undergoing CXL for KC	CXL vs. no CXL	Found moderate-quality evidence supporting efficacy of CXL in stabilizing KC and improving corneal curvature, safety outcomes also reported	Good
Urgacz et al. 2015 [104]	Narrative review	Allergy sufferers wearing contact lenses	Summarizes literature on contact lens complications in allergic patients	Identifies increased risk of corneal hypoxia, hyperemia, microinjuries, tear-film disruption, and hypersensitivity reactions (5% in soft lenses, 2%–5% in rigid lenses)	Fair
Wisse et al. 2015 [105]	Systematic review	KC patients evaluated across multiple cytokine expression studies	Analysis of soluble and cellular mediators in tears and corneal tissue	Confirms evidence of elevated inflammatory markers (IL‑1β, IL‑6, TNF‑α), but highlights methodological inconsistencies across studies	Good
Bae et al. 2016 [106]	Prospective interventional	Dry eye patients resistant to conventional therapy	Daily oral vitamin D supplementation vs. baseline	Improvement in tear stability and symptom scores in vitamin D insufficient patients	Fair
Baudouin et al. 2016 [107]	Narrative review	Dry eye and MGD patients via literature review	N/A	Refines the “vicious circle” model of dry eye, emphasizing MGD’s role in perpetuating inflammation and tear instability	Good
Bykhovskaya et al. 2016 [108]	Review	Genetic studies of KC	N/A	Details candidate genes (e.g., VSX1, TGFBI, COL5A1) and mechanisms, discussing future directions in genetic analysis	Fair
Gatinel 2016 [109]	Perspective commentary	N/A	N/A	Argues that eye rubbing is essential (“sine qua non”) for KC development, endorsing mechanical trauma theory	Fair
Huang et al. 2016 [110]	Randomized, double-blind, placebo-controlled trial	Dry eye patients	Oral antioxidant supplement vs. placebo over weeks	Significant improvement in tear stability and symptom scores in the treatment arm	Good
Ionescu et al. 2016 [111]	Cross-sectional observational	KC patients vs. controls	Tear inflammatory biomarkers measured and profiled	Characterized KC tear microenvironment showing elevated MMP‑9, IL‑6, IL‑8	Good
Lanza et al. 2016 [112]	Diagnostic	Dry eye patients and controls	Point-of-care tear MMP-9 test vs. clinical DED metrics	Test showed sensitivity ~ 85% and specificity ~ 94%, correlating with severity and osmolarity	Good
Mandathara et al. 2016 [113]	Pilot observational study	KC patients (n ≈ 40)	Corneal sensitivity (Cochet–Bonnet) vs. clinical metrics	Reduced sensitivity correlated with higher disease severity and thinner central corneas	Fair
Pahuja et al. 2016 [114]	Comparative molecular case-control (cone apex vs. periphery)	KC corneal epithelial (n = 66) and stromal tissue compared with controls (n = 23)	qPCR of ECM and inflammatory gene expression in cone vs. periphery regions	Cone apex showed elevated TNF-α, IL-6, MMP‑9, decreased LOX and Collagen IVα1; expression correlated with corneal curvature and breaking of Bowman’s layer	Good
Shen and Ma 2016 [115]	Observational case-control	44 postmenopausal dry-eye patients vs. 22 controls	Serum estradiol and tear MMP‑2/9 levels correlated with tear function metrics	Higher estradiol was associated with increased MMP‑2/9 activity and lower Schirmer scores	Good
Akçay et al. 2017 [116]	Prospective cohort	Progressive KC patients undergoing accelerated CXL	Evaluation of tear function and ocular surface before/after CXL	Transient reduction in tear stability and surface integrity immediately post-CXL, with recovery by 3 months	Good
Arbab et al. 2017 [117]	Case-control genetic expression study	KC patients vs. healthy controls	TNF-α gene promoter polymorphism analysis and corneal tissue expression profiling	Identified TNF-α SNP linked to increased expression and KC risk	Good
Benitez-Del-Castillo et al. 2017 [118]	Consensus proceedings	Dry eye patients assessed for vision-related outcomes	Reviews correlating VA impairment and quality-of-life impacts	DED significantly lowers vision-related quality of life; standardized recommendations	Fair
Bron et al. 2017 [119]	Consensus report (DEWS II Pathophysiology)	Synthesizes a wide literature on DED mechanisms	N/A	Comprehensive framework on tear film dysfunction, inflammation, neurosensory abnormalities, and ocular surface damage	Good
Golebiowski et al. 2017 [120]	Case-control clinical study	Postmenopausal women with/without MGD	Serum estrogen levels correlated with meibomian gland function	Endogenous estrogen levels did not significantly correlate with MGD severity	Good
Khaled et al. 2017 [121]	Cross-sectional molecular-histologic analysis	KC corneal buttons vs. normal specimens	Multimodal evaluation of tissue for cytokine, MMP, and ECM protein expression	Highlighted elevated inflammatory mediators and disrupted collagen/fibronectin architecture in KC	Good
Martinez-Revelles et al. 2017 [122]	Experimental cardiovascular study	Animal models of hypertension	Role of LOX signaling in arterial stiffness via p38MAPK pathway	LOX upregulation contributed to vascular oxidative stress and elastin abnormalities via p38MAPK signaling	Good
Shetty et al. 2017 [123]	Cross-sectional observational	Indian KC patients vs. controls	Tear cytokine profiling	Confirmed elevated IL-6, IL-17, and TNF‑α in KC tears; supported an inflammatory phenotype in this population	Good
Sobrino et al. 2017 [124]	Case-control with molecular profiling	40 bilateral KC patients vs. 20 healthy controls	Flow cytometry analysis of TLR2 and TLR4 expression in monocytes and neutrophils; serum levels of IL‑1β, IL‑6, TNF‑α, MMP‑9, NF-κB also measured	Significantly higher TLR2 and TLR4 expression levels in KC patients (*P* < 0.0001), with correlations to elevated pro-inflammatory markers	Good
White et al. 2017 [125]	Histological comparative study	Explanted post-keratoplasty KC corneal buttons vs. normal donor tissue	Electron microscopy to map elastic microfibril distribution	Keratoconic stromas showed absence of microfibrils anterior to Descemet’s membrane and abnormal concentration below epithelium; contrasts with normal distribution	Good
Alio Del Barrio et al. 2018 [126]	Interventional proof-of-concept	15 patients with advanced KC unsuitable for CXL	Implantation of decellularized stromal laminas, with or without recellularization with autologous stem cells	Improved topography, thickness, and visual acuity over 12 months; stem cell-implemented group showed better integration	Good
Bamdad et al. 2018 [127]	Cross-sectional biochemical study	45 advanced KC patients vs. 45 controls	Serum levels of zinc, calcium, magnesium, iron, copper, selenium	KC patients had significantly lower serum zinc and copper (*P* < 0.05), suggests systemic mineral imbalance	Good
Cifariello et al. 2018 [128]	Prospective comparative study	60 KC patients randomized to epi-off or epi-on CXL	Standard vs. transepithelial CXL assessed over 2 years	Both groups showed stable topography; epi-on had less haze but marginally less flattening	Good
Foulsham et al. 2018 [129]	Narrative immunology review	Literature on ocular immune regulation	N/A	Explores how innate and adaptive immunity maintain ocular clarity, implications for inflammatory disease	Fair
Ionescu et al. 2018 [130]	Cross-sectional family-based study	KC patients, first-degree relatives, and controls (n unspecified)	Tear cytokine profiling	Both KC patients and their relatives exhibited elevated tear cytokines (IL‑6, MMP‑9), suggesting predisposition	Good
Kessal et al. 2018 [131]	Observational	DED patients vs. controls	Conjunctival impression cytology with HLA‑DRA and HLA‑DRB1 transcript analysis	Increased HLA expression correlated with disease severity	Good
Seen and Tong 2018 [132]	Narrative review	Literature on oxidative stress in DED	N/A	Highlights ROS damage in the ocular surface and therapeutic targets in antioxidants	Fair
Sharif et al. 2018 [133]	In vitro/ex vivo mechanistic study	Human corneal fibroblasts from KC patients	CXL-treated vs. untreated stromal cells	CXL enhanced collagen and ECM protein expression, improved cell stiffness, and mitochondrial function	Good
Yang et al. 2018 [134]	Prospective interventional	32 dry eye patients with vitamin D deficiency (< 50 nmol/L) assessed at baseline and after 2 months of oral vitamin D	Daily vitamin D supplementation	Serum vitamin D increased by ~ 29 nmol/L; significant reductions in dry eye symptoms, ocular surface staining, and improved tear quality; IL‑6 remained unchanged	Good
You et al. 2018 [135]	Comparative RNA‑Seq analysis	Corneal epithelium from KC patients (pre‑CXL) and myopic PRK controls	Transcriptome comparison	KC samples exhibited significant downregulation of genes involved in Hedgehog, Wnt, Notch1, including plasmolipin; Notch1 and plasmolipin protein reductions confirmed (*P* < 0.05)	Good
Arita et al. 2019 [136]	Prospective interventional clinical	Patients with refractory MGD (n ≈ 40)	Single session IPL therapy	Improved meibum expressibility, lipid layer thickness, tear breakup time, and symptom scores sustained over 3 months	Good
Borchman 2019 [137]	Experimental thermal biophysics study	Meibum samples analyzed under varying heat regimes	Temperature variations to determine optimal liquefaction for therapy	Identified 40–45 °C as the optimal temperature range for effective meibum melting	Good
di Martino et al. 2019 [138]	Narrative review	Literature on MMP roles in KC	N/A	Discussed MMP overactivity and ECM degradation as drivers of stromal weakening	Fair
Di Zazzo et al. 2019 [139]	Cross‑sectional biochemical clinical	Adults with VKC (n ≈ 30)	Tear cytokine and biomarker analysis	Elevated levels of IL‑8, eotaxin, and ECP correlated with clinical severity	Good
Herbaut et al. 2019 [140]	Narrative review	Literature on tear film on ocular optical quality	N/A	Highlighted the relationship between tear-film stability, osmolarity, and visual quality (e.g., scatter, aberrations)	Fair
Jabbehdari et al. 2019 [141]	Retrospective case series	Severe atopic keratoconjunctivitis patients with ocular surface disease	Systemic/topical immunomodulation, amniotic membrane grafting	Visual acuity and ocular surface restoration achieved in most cases	Fair
Jie et al. 2019 [142]	Cross‑sectional clinical	50 dry eye patients and 50 healthy controls	Blinking patterns measured by infrared oculography; correlated with dry eye scores	Incomplete blinking rates were significantly higher in dry eye and correlated with symptom severity	Good
Khairy et al. 2019 [143]	Medium-term randomized controlled trial	60 eyes post-refractive surgery or after penetrating keratoplasty with corneal ectasia	Accelerated CXL vs. standard CXL	Both modalities halted ectasia progression; accelerated CXL led to faster recovery and similar long-term outcomes over 2 years	Good
Najmi et al. 2019 [144]	Literature review	24 peer-reviewed studies on KC and eye rubbing	N/A	Strong link: eye rubbing causes keratocyte thinning; risk proportional to rubbing duration and force. Recommends avoiding rubbing by treating the itch and dryness	Good
Shetty et al. 2019 [145]	Prospective observational molecular/biochemical	83 KC eyes vs. 42 controls	Corneal epithelial cells from the cone vs. the peripheral regions	Cone cells showed increased apoptosis, reduced differentiation, EMT signatures; epithelial behavior varies by region—implications for personalized treatment	Good
Shinde et al. 2019 [146]	Proteomic profiling	Unpooled corneas from KC patients	High-resolution tear and tissue proteomics	Identified molecular KC subtypes with distinct protease and inflammatory profiles	Good
Tamhane et al. 2019 [147]	Narrative review	Overview of ocular biomarker challenges	N/A	Examines tear and ocular tissue biomarkers—emphasizes standardization challenges and translational possibilities	Fair
Ahuja et al. 2020 [148]	Narrative review	Reports on IgE, allergy, and eye rubbing in KC	N/A	Highlights the role of the allergy-immunity axis with IgE and rubbing behaviors in KC pathogenesis	Fair
Bang et al. 2020 [149]	Laboratory validation study	Tear samples from dry eye/KC contexts	In situ MMP‑9 immunoassay validated against ELISA	Strong concordance and reliability—supports its use as a point-of-care wet biomarker	Good
Doroodgar et al. 2020 [150]	Case series	KC patients receiving stromal lenticule implantation	Customized allogenic lenticule grafts for corneal reinforcement	Increased corneal thickness and improved vision at short follow-up	Good
Fuller and Wang 2020 [151]	Retrospective cohort	KC patients fitted with scleral lenses	Assessment of safety, fit success, comfort, and visual acuity	High fitting success, good VA outcomes, occasional mild complications (e.g., redness)	Good
Hsueh et al. 2020 [152]	In vitro cellular study	Cultured human corneal endothelial cells under oxidative stress	Ascorbic acid treatment vs. untreated	Ascorbic acid reduced apoptosis, stabilized autophagic flux under oxidative stress	Good
Jamerson et al. 2020 [153]	Narrative review	Review of MMP‑9 role across ocular surface disorders	N/A	MMP‑9 levels correlate with severity across diseases; point-of-care MMP‑9 suggested as a useful clinical biomarker	Fair
Kenny et al. 2020 [154]	Narrative review	Contact lens users and patients with ocular prostheses, sutures, or buckles	Analysis of mechanical vs. immunologic causation	GPC arises from mechanical trauma to the superior tarsal conjunctiva, plus an immune response to lens deposits	Fair
Kheirkhah et al. 2020 [155]	Randomized controlled trial	42 patients with refractory obstructive MGD and lid tenderness	MG probing + sulfacetamide/prednisolone ointment; MG probing + lubricating ointment; Sham probing + lubricating ointment	MG probing + steroid produced significant symptom improvement (OSDI, SANDE) at 4 weeks; MG probing + lubricant improved SANDE only; Sham group had no change in symptoms	Good
Liu et al. 2020 [156]	Observational	Patients with pterygium, dry eye, or both	Tear VEGF concentrations evaluated	Elevated VEGF correlated with both pterygium and dry eye features, suggesting a shared inflammatory pathway	Fair
Nemeth et al. 2020 [157]	Cross-sectional	KC patients vs. controls	OSDI symptom scoring and ocular surface temperature mapping	Increased OSDI scores and distinct ocular thermographic patterns in KC	Fair
Pellegrini et al. 2020 [158]	Narrative review	Literature on diet and supplements in ocular surface conditions	Omega‑3, vitamins, and antioxidants	Nutrient supplementation (e.g., omega‑3, vitamin D, and vitamin A) shows potential benefits in dry eye and related disorders	Fair
Shinde et al. 2020 [159]	Transcriptomic analysis	Corneal samples from two KC patient groups and controls	RNA sequencing	Identified biomarkers; reduced NRF2 antioxidant pathway expression in KC	Good
Situ et al. 2020 [160]	Observational case series	Contact lens wearers	Corneal sensitivity measured morning vs. evening	Reduced sensitivity in the evening correlates with increased lens discomfort	Fair
Tashbayev et al. 2020 [161]	Concise narrative review	Literature on IPL therapy for MGD	IPL	Supports IPL improving meibum expressibility, symptom relief, and tear stability, with low adverse effects	Fair
Wei et al. 2020 [162]	Narrative review	Broad review of the LOX family across tissues	N/A	LOX enzymes implicated in ECM development and stiffness across organs, including potential corneal relevance	Fair
Bai et al. 2021 [163]	Observational	Patients with MGD and controls	Imaging of tear lipid-layer dynamics (Tearscope/OCT)	MGD patients had unstable lipid films, dynamic thinning patterns, and correlated with symptoms	Good
D'Souza et al. 2021 [14]	Prospective case-control	32 KC patients (51 eyes, various grades) and 15 healthy controls (23 eyes)	Ocular surface immune cell profiling via flow cytometry (leukocytes, neutrophils, NK cells, γδ T cells); tear multiplex ELISA assessing 50 soluble factors	Elevated activated neutrophils, NK cells, γδ T cells; higher tear levels of IL‑1β, IL‑6, IL‑17A, TNF-α, IFNs, EPO, TGF‑β1, MMP‑2, IgE, and others; lower IL‑1α and IL‑9. Immune markers correlated with disease grade, eye rubbing, keratometry, and pachymetry	Good
Ding et al. 2021 [164]	Observational cross-sectional	Healthy adults	Infrared ocular thermography to measure ocular surface cooling rate, correlated with tear film characteristics and maximum inter-blink period	Faster cooling linked to thinner lipid layer and shorter inter-blink periods, suggesting tear film evaporation & blink interplay	Fair
Foster et al. 2021 [165]	Transcriptomic + immunohistochemical analysis	Progressive KC corneal samples	RNA‑Seq and tissue staining	Altered WNT10A expression detected in epithelium and Bowman’s layer, indicating disrupted corneal homeostasis mechanisms	Good
Hall et al. 2021 [166]	Retrospective cohort	Stevens-Johnson syndrome/toxic epidermal necrolysis patients treated with systemic cyclosporine	Pre- and post-treatment ocular assessments	Systemic cyclosporine associated with reduced ocular inflammation, lower incidence of chronic keratitis amidst acute SJS/TEN	Fair
Hao et al. 2021 [167]	Multi-level gene enrichment review	Published KC gene expression studies	N/A	Mapped gene-enriched pathways—ECM remodeling, oxidative stress, inflammation, immune signaling (e.g., NF‑κB, TGF‑β, Wnt)	Fair
Ng et al. 2021 [168]	Systematic review	Patients undergoing transepithelial vs. epi-off CXL	Transepithelial corneal collagen cross-linking (TE-CXL) vs. Epi-off CXL	TE-CXL has lower efficacy in halting progression and flattening the cornea; similar safety profiles; moderate-quality evidence	Good
Tunç et al. 2021 [169]	Narrative review	Literature on ocular surface microbiota in KC	N/A	Emerging evidence suggests microbiome differences (e.g., reduced beneficial commensals), potentially contributing to inflammation and epithelial disruption	Fair
Yu et al. 2021 [170]	Narrative review	Literature on immune and inflammatory mechanisms in dry eye	N/A	Highlights roles of ocular antigen-presenting cells, Th17/IL-17 pathway, pyroptosis, and ocular-surface-nerves in DED pathophysiology	Fair
Zhang et al. 2021 [171]	Meta-analysis of case-control and cross-sectional studies	Multiple KC studies measuring tear cytokines	Tear levels vs. control eyes	Pooled results show significantly higher IL‑6, IL‑8, and TNF‑α in KC tears; significant heterogeneity noted	Good
Ahn et al. 2022 [172]	Cross-sectional clinical study	Patients with MGD and/or aqueous deficiency vs. controls	Clinical signs (MG dropout, tear meniscus height) and FRD metrics	FRD strongly associated with moderate-to-severe MGD (OR: ~ 12) and AD; higher gland dropout, lower tear meniscus height, shorter NIKBUT-1 (*P* < 0.001); highlights lipid dysfunction’s central role	Good
Akiyama et al. 2022 [173]	Retrospective observational	31 KC eyes, newly using rigid gas-permeable (RGP) lenses, no lens use in the previous month	AS-OCT keratometry and pachymetry before vs. after ≥ 1 month of RGP wear	Average K decreased by 1.05 ± 1.92 D (*P* < 0.01); Kmax decreased 1.65 ± 4.20 D (*P* = 0.04); greater effects in severe KC; no change to central or thinnest corneal thickness	Good
Caban et al. 2022 [174]	Narrative review	Literature summary on MMPs and TIMPs	N/A	Discusses MMP/TIMP imbalance contributing to corneal and ocular surface diseases, including KC and dry eye	Fair
Koh et al. 2022 [175]	Narrative review	Compilation of ocular surface and tear film measurement techniques	Evaluation techniques (OCT, osmolarity, biomarkers) compared in diagnostic accuracy	Reviews current approaches (imaging, osmolarity, lipid evaluation, sensors) for dry eye diagnosis	Fair
Labib and Chigbu 2022 [176]	Narrative review	Allergic conjunctivitis clinical interventions literature	Various therapeutic targets – antihistamines, mast cell stabilizers, biologics	Evaluates antihistamines, mast-cell stabilizers, steroids, immunotherapies, and biologics	Fair
Lasagni Vitar et al. 2022 [177]	Cross-sectional	Nutrient/metabolic profiling in KC patients	Nutritional/metabolic markers compared to healthy controls	Identified alterations in amino acids, lipids, and energy metabolites, suggesting impaired metabolic homeostasis in KC	Good
Lupasco et al. 2022 [178]	Observational	Corneal epithelium from 22 early KC, 6 advanced KC, and 23 controls	RT-PCR of keratin differentiation and oxidative stress genes; immunofluorescence for active NRF2	Early KC showed downregulation of NRF2, HMOX1/2, keratin genes; advanced KC had reduced SPRR2A/HMOX1; reduced NRF2 staining confirmed	Good
Martinez-Perez et al. 2022 [179]	Observational	KC patients	Meibomian gland assessment (meibography, expressibility, tear film tests)	Significant MGD was present in KC patients, associated with tear instability	Good
Napolitano et al. 2022 [180]	Comparative observational	KC patients treated with epi-on vs. epi-off iontophoresis CXL	Topographic outcomes monitored over follow-up	Both techniques showed efficacy, with epi-off achieving slightly better flattening but more haze	Good
Navel et al. 2022 [181]	Systematic review & meta-analysis	Dry eye patients’ oxidative/antioxidative biomarker studies	Comparison of stress markers across patient/control arms	Revealed elevated oxidative stress markers (e.g., MDA) and reduced antioxidants (e.g., SOD, GSH-Px) in DED vs. controls	Good
Niazi et al. 2022 [182]	Case series/proof-of-concept	Ectatic corneal disorder patients (e.g., KC) receiving stromal lenticule implantation	Corneal stromal augmentation using decellularized lenticules	Improved corneal thickness and biomechanical strength; visual outcomes appear promising	Good
Suzuki et al. 2022 [183]	Cross-sectional	Young/elderly healthy subjects and MGD patients	Meibum lipid composition (non-polar vs. polar) and symptom survey	With aging and MGD, non-polar lipids (cholesterol esters) decreased, while polar lipids increased (*P* < 0.05); blurred vision correlated with low non-polar and high polar lipid levels	Good
Vernhardsdottir et al. 2022 [184]	Narrative review	Literature on antibiotic treatments for MGD and blepharitis-related dry eye	Evaluation of oral/topical antibiotics for bacterial/inflammatory control	Supports azithromycin and doxycycline as effective in moderate to severe cases, highlighting resistance concerns	Fair
von Ahrentschildt et al. 2022 [185]	Cross-sectional	Contact lens wearers vs. non-wearers	Meibography, gland dropout, tear film, symptom assessment	Contact lens wear was associated with greater gland tortuosity and dropout, reduced tear stability, and increased symptoms	Good
Zhang et al. 2022 [186]	In vitro	Human corneal epithelial cell cultures	Hyperosmolar stress (> 450 mOsm) with/without TNF‑α or MMP inhibitors	Tight-junction disruption via TNF‑α–mediated MMP upregulation; inhibitors preserved barrier integrity	Good
Zhou et al. 2022 [187]	Cross-sectional observational	KC patients vs. matched controls	Tear film lipid layer thickness via interferometry	KC patients had significantly thinner lipid layers (*P* < 0.05), correlating with ocular surface symptoms	Good
Byambajav et al. 2023 [188]	Cross-sectional	Type 2 diabetics with/without dry eye vs. healthy controls	Tear biomarker profiling (lipids, osmolarity) and quality-of-life questionnaire	Dry eye diabetes patients showed distinct biomarker profiles and worse vision-related quality-of-life scores	Good
Cardona et al. 2023 [189]	Observational	N/A	Blink regularity tracked via high-speed video; ocular surface exposure analyzed via fluorescein	Irregular blinking (e.g., incomplete, rapid) doubled ocular surface exposure time compared to regular blinks	Fair
Dammak et al. 2023 [190]	Narrative review	Ocular surface diseases grouped by oxidative stress evidence	Antioxidant and diagnostic marker discussion	Highlights key oxidative biomarkers and potential antioxidant therapies for dry eye, KC, and chemical injuries	Fair
Doroodgar et al. 2023 [191]	Book chapter narrative	Literature on complications in modern penetrating keratoplasty	Corneal transplant procedures, patient profiles	Discusses challenges like graft rejection, astigmatism, wound issues, infection, and innovation strategies	Fair
Gomes et al. 2023 [192]	TFOS lifestyle consensus report	Broad literature on elective medications/procedures and ocular surface health	Elective topical/systemic medications and non-essential surgical procedures	Non-urgent interventions (e.g., cosmetic eyelid surgery, refractive procedures like SMILE) can disrupt the tear film and ocular surface—example: SMILE causes more early-month vision disturbances but less long-term dry eye than LASIK	Good
Gorimanipalli et al. 2023 [193]	Narrative review	Draws from clinical/lab studies on sex and endocrine hormones in DED	Covers androgen, estrogen, progesterone, insulin, and thyroid influences	Highlights androgen deficiency [e.g., menopause, polycystic ovary syndrome (PCOS), insulin resistance] as key driver of DED via meibomian/tear dysfunction; also discusses estrogen's mixed effects and emerging topical insulin therapies	Good
Hage et al. 2023 [194]	Observational	153 patients with KC or ocular surface disease	Self-report evaluations using addiction scales (Goodman, CAGE)	81.7% reported eye rubbing; 63% met addiction criteria; higher rates of psychiatric history; supports addictive-like behavior with peripheral/central sensitization cycle	Good
Hu and Trief 2023 [195]	Narrative review	Literature on LSCD and ocular surface disease	N/A	Reviews causes (autoimmune, chemical, etc.), clinical features, and management strategies, including stem cell transplantation	Fair
Jiang et al. 2023 [196]	Genetic co-localization study	Genome-wide association studies (GWAS)-identified corneal resistance factor loci with GTEx e/sQTL data	Bioinformatic colocalization of trait loci with GTEx e/sQTLs	Identified candidate genes (e.g., COL5A1 alternative splicing) influencing postnatal stromal weakening—potential mechanisms underlying KC	Good
Jones et al. 2023 [197]	Consensus synthesis	Literature on contact lens use and ocular surface health	N/A	Demonstrates that contact lenses disrupt the tear film, induce inflammation; emphasizes lens materials, hygiene, wear schedules, and lens care as modifiable risk factors	Good
Loureiro et al. 2023 [198]	Prospective	Primary Sjögren syndrome dry eye patients	Treated medically, epithelial thickness via OCT before and after	Central and inferior epithelial thinning reversed after therapy, concurrent improvement in symptoms	Good
Marques et al. 2023 [199]	Cross-sectional observational	KC patients vs. controls	Corneal epithelial cell collection and multiplex cytokine analysis	KC epithelium showed elevated IL‑6, IL‑8, TNF‑α, MMP‑9; levels correlated with cone severity	Good
Mazharian et al. 2023 [200]	Prospective cohort with behavioral intervention	“At risk” eye rubbers with keratoplasty risk	Counseling and cessation of eye rubbing	Reduction in progression rate by ~ 45%; improved corneal tomography stability over 1–2 years	Good
Niazi et al. 2023 [201]	Nationwide registry	KC cases and matched population controls in the registry	LOX gene SNP analysis	Two LOX polymorphisms (rs1800449, rs2288393) significantly associated with KC risk (OR ~ 1.4–1.6)	Good
Regueiro et al. 2023 [202]	Narrative review	Literature on the impact of lactoferrin use on the ocular surface	Biological and therapeutic analysis	Reviews anti-microbial, anti-inflammatory, wound‑healing roles; supports its use in DED, contact lens care, and postoperative healing	Fair
Rocha-de-Lossada et al. 2023 [203]	Multicenter case-control	38 naïve KC patients vs. 167 healthy controls	16S rRNA sequencing of ocular surface microbiota	KC patients exhibited lower microbiome diversity, with a unique presence of Pelomonas and Ralstonia, suggesting that ocular microbiota alterations may contribute to KC pathophysiology	Good
Roshandel et al. 2023 [204]	Narrative review	Genetic predisposition literature across ocular surface disorders	N/A	Summarizes known gene variants (e.g., LOX, TGFB1, TIMP3) and discusses prospects for gene-based therapies in corneal dystrophies and KC	Fair
Sarma et al. 2023 [205]	Systematic review and meta-analysis of randomised controlled trials (RCTs)	48 RCTs comparing CXL vs. control or different CXL protocols	CXL vs. control/placebo	Conventional epi-off CXL significantly improved Kmax (~ − 1.78 D at 1 year), BCVA, and central corneal thickness vs. control. Transepithelial methods were less effective in the long term. Safety profile acceptable	Good
Tunç et al. 2023 [206]	Observational	KC patients vs. controls	Next-generation 16S rRNA sequencing of corneal swabs	Identified distinct microbial community structures in KC corneas, with reduced beneficial commensals and increased pathogens	Good
Verges et al. 2023 [207]	Proof-of-concept	MGD patients vs. controls	Conjunctival fornix aspirates analyzed for transcriptomic signatures	Upregulation of innate immunity and lipid metabolism genes (e.g., TLR4, CD36), supporting a pathophysiological link between MGD and ocular surface inflammation	Fair
Weng et al. 2023 [208]	Narrative review	Laboratory and clinical literature on artificial tear ingredients	N/A	Critically evaluates the physicochemical effects of components like hyaluronate, salts, osmoprotectants, and lipids. Recommends formulation choices based on epitheliotropic and osmoprotective properties	Fair
Acar Eser et al. 2024 [209]	Prospective	KC patients undergoing conventional epi-off CXL	Measured tear cytokine and chemokine levels pre- and 6 months post-CXL	Significant post-CXL reductions in IL‑6, IL‑8, TNF‑α, and MMP‑9. Suggests therapeutic anti-inflammatory effects of CXL beyond structural reinforcement	Good
Alam et al. 2024 [210]	Single-cell RNA sequencing	Mice or human corneal samples after short-term experimental dry eye	scRNA-Seq profiling of immune cell populations on the cornea	Identified early expansion of inflammatory macrophages, dendritic cells, and Th17 cells. Maps cellular responses involved in DED onset and resolution	Good
Chan et al. 2024 [211]	Systematic review	Adults (23–84 years), human tears/serum/cornea; rabbit & mouse models; n = 12–122	VEGF/VEGFR levels in tears, serum, plasma, cornea; compared with healthy controls or non-diseased eyes; some treated vs. untreated animal models	VEGF concentration/expression; correlation with CNV, DED, AED, keratitis; links to IL-6/IL-8; response to anti-VEGF therapy	Fair
Hsu et al. 2024 [212]	Genome-wide polygenic risk score (PRS) study	Large East Asian population cohorts	Calculated DED risk via PRS models; associated PRS with clinical DED incidence	PRS stratified risk effectively (top decile OR ~ 2.5); supports genetic risk prediction approach	Good
Kaur et al. 2024 [213]	Case-control	10 KC corneas vs. three healthy controls	mNGS on corneal epithelium	No significant difference in microbial read counts (KC avg 530 vs. control 622; *P* = 0.29); Proteobacteria equally dominant (~ 72% vs. 79%). Concludes no evidence of chronic infection in KC	Good
Lv et al. 2024 [214]	Narrative review	Research on nanomedicine therapies for dry eye	Nanocarriers loaded with drugs/antioxidants	Highlights nanoparticle systems (e.g., liposomes, hydrogels, micelles), improving drug retention, bioavailability, and therapeutic efficacy in dry eye	Fair
Santos et al. 2024 [215]	Case-control	KC patients vs. controls	Ocular surface tissue analysis (e.g., conjunctival/corneal cytology)	KC tissues showed higher expression of IL‑6, IL‑8, TNF‑α, MMP‑9—supporting ongoing surface inflammation in KC	Good
Shukla et al. 2024 [216]	Narrative review	Broad discussion on corneal epithelial thickness	Cites the use of OCT in conditions like dry eye, KC, and refractive surgery	Emphasizes epithelial mapping as key to early disease detection, healing assessment, and refractive integrity	Fair
Kumar et al. 2025 [217]	Prospective case-control	62 KC eyes vs. 21 healthy controls	16S rRNA sequencing of ocular swabs; tear ELISA for 52 cytokines; flow cytometry for 11 immune-cell types	KC eyes exhibited distinct microbiome signatures (e.g., increased Microbacterium, Cutibacterium) matching disease grade; significant cytokine elevation (IL‑6, IL‑21, MMP‑2) and immune cell shifts (CD45 + , CD66b), indicating microbiome-driven immune dysregulation	Good

*AS-OCT* = anterior segment optical coherence tomography; *BDNF* = brain-derived neurotrophic factor; *BCVA* = best-corrected visual acuity; *BMP* = bone morphogenetic protein; *CD* = cluster of differentiation (e.g., CD45 + , CD66b); *COL5A1* = collagen type V alpha 1 chain; *CXL* = corneal cross-linking; *DED* = dry eye disease; *DES* = dry eye syndrome; *ECP* = eosinophil cationic protein; *EGF* = epidermal growth factor; *ELISA* = enzyme-linked immunosorbent assay; *EMT* = epithelial-mesenchymal transition; *epi-on* = epithelium-on; epi-*off* = epithelium-off; *eQTLs* = expression quantitative trait loci; *FGF* = fibroblast growth factor; *FRD* = friction-related disease; *GDNF* = glial cell line-derived neurotrophic factor; *GPC* = giant papillary conjunctivitis; *GSH-Px* = glutathione peroxidase; *GTEx* = genotype-tissue expression; *HMOX1/2* = heme oxygenase 1/2; *HRT* = hormone replacement therapy; *ICAM-1* = intercellular adhesion molecule 1; *IGF-I* = insulin-like growth factor I; *IL* = interleukin; *IOP* = intraocular pressure; *IPL* = intense pulsed light; *Kmax* = maximum keratometry; *KC* = keratoconus; *KGF* = keratinocyte growth factor; *LASIK* = laser-assisted in situ keratomileusis; *LOX* = lysyl oxidase; *LSCD* = limbal stem cell deficiency; *LTC4* = leukotriene C4; *MGD* = meibomian gland dysfunction; *MMP* = matrix metalloproteinase; *mNGS* = metagenomic next-generation sequencing; *NGF* = nerve growth factor; *NIBUT* = noninvasive break-up time; *NIKBUT* = noninvasive keratograph break-up time; *NRF2* = nuclear factor erythroid 2–related factor 2; *OCT* = optical coherence tomography; *OR* = odds ratio; *OSDI* = ocular surface disease index; *PDGF* = platelet-derived growth factor; *PMMA* = polymethylmethacrylate; *PRK* = photorefractive keratectomy; *qPCR* = quantitative polymerase chain reaction; *ROS* = reactive oxygen species; *RT-PCR* = reverse transcription polymerase chain reaction; *SANDE* = symptom assessment in dry eye; *scRNA-Seq* = single-cell RNA sequencing; *SMILE* = small incision lenticule extraction; *SNP* = single nucleotide polymorphism; *SOD* = superoxide dismutase; *SPRR2A* = small proline rich protein 2A; *sQTLs* = splicing quantitative trait loci; *TBUT* = tear break-up time; *TFOS* = Tear Film and Ocular Surface Society; *TGF-β* = transforming growth factor-beta; *TGFBI* = transforming growth factor beta induced; *Th17* = T helper 17 cells; *TIMP* = tissue inhibitor of metalloproteinases; *TLR* = toll-like receptor; *TNF-α* = tumor necrosis factor-alpha; *UVA* = ultraviolet A; *VEGF* = vascular endothelial growth factor; *VKC* = vernal keratoconjunctivitis

## Discussion

### Ocular surface microenvironment changes in KC

A brief review of how each marker contributes to KC development is presented in Table 2.

**Table 2:** Ocular surface and biochemical changes in keratoconus (KC)

Category (Subsection)	Biomarker/feature	Observed change	Additional comments
1. Inflammation: tear fluid cytokines	IL-1β [171, 215]	Increased	Elevated in KC tears; drives ECM degradation and keratocyte apoptosis
	IL-5 [14, 67, 111, 123, 130, 209]	Increased	Pro-inflammatory cytokine promoting tissue remodeling
	IL-6 [117, 124, 171, 215]	Increased	Enhances MMP production and ECM breakdown; key mediator of progression
	IL-8 [171, 215]	Increased	Recruits inflammatory cells; contributes to KC pathogenesis
	TGF-β [215]	Increased	Involved in ECM remodeling and fibrosis, contributing to corneal thinning
	CCL5 [62, 215]	Variable	Inconsistent findings are likely due to methodological differences
	IL-17 [62]	Increased	Associated with protease upregulation and tissue degradation
	TNF-α [62, 171]	Variable	Plays a role in MMP activation; levels vary by detection method
	IL-12 [62]	Decreased	Suggests an altered Th1 immune response
	IFN-γ [62]	Decreased	Indicates diminished Th1 cytokine activity
	IL-4/IL-13 [62, 171]	Decreased/Unchanged	No consensus on Th2 changes
	IL-10 [171]	Unchanged	Anti-inflammatory cytokine remains stable
2. Inflammation: MMP cascades	MMP-9 [101, 138, 174]	Increased	Key ECM-degrading enzyme; highly upregulated in KC tears and epithelium; potential screening marker
	MMP-1 [138]	Increased	Contributes directly to collagen breakdown
	MMP-3 [138]	Increased	Acts with other MMPs to remodel the ECM
	MMP-7 [138]	Increased	Participates in proteolytic activity; its precise role is less defined
	MMP-13 [138]	Increased	Likely involved in collagen breakdown and corneal thinning
	MMP-2 [138]	Variable	Some studies report increases; others show no significant change
	TIMP-1 [153]	Decreased	Reduced inhibition leads to unchecked MMP activity
3. LOX enzyme activity	LOX activity [97]	Decreased	Reduced enzyme activity decreases collagen cross-linking, weakening the corneal structure
4. Sex hormones	Androgen receptor expression [177]	Increased	May modulate inflammatory/MMP responses affecting corneal biomechanics
	Estrogen receptor expression [177]	Increased	Likely influences corneal structure and local inflammation
	Progesterone receptor expression [177]	Decreased	Indicates altered hormonal regulation in KC
	Hormonal influences (puberty/pregnancy) [97, 177]	Associated with increased progression	Fluctuations (e.g., during puberty or post-pregnancy) can enhance MMP production and ECM remodeling
5. Growth factors	NGF [105]	Increased	Elevated levels may alter corneal innervation and epithelial metabolism
	VEGF [22, 58]	Variable	Conflicting reports; its role in vascular permeability and MMP induction remains under investigation
6. Genetic alterations	LOX gene, CAST, SLC4A11, TGFBI, MPDZ-NF1B, WNT10A, IL-1α, IL-1β, IL-1RN, microRNA184, HGF, RAB3-GAP1, DOCK9, COL4A3, COL4A4, COL5A1, FNDC3B, SOD1, ZNF469, ZEB1, VSX1, FOXO1 [59, 93, 108, 131, 147, 167, 212]	Increased susceptibility	Polymorphisms in these genes (including the LOX gene) predispose to KC via defective collagen cross-linking, impaired ECM integrity, increased oxidative stress, and dysregulated inflammation
7. Molecular pathways & protein expression	eIF/mTOR pathway proteins [146]	Increased	Dysregulated signaling alters protein synthesis and ECM turnover
	ECM & acute-phase proteins [146]	Decreased	Suggests impaired tissue repair and chronic degeneration
	Wnt signaling (e.g., Wnt10A) [135, 165]	Decreased	Under-expression may weaken epithelial–stromal interactions
	NRF2 (antioxidant regulator) [146, 159]	Decreased	Loss impairs antioxidant defenses, increasing oxidative damage
	KEAP1 [159]	Increased	Elevated levels further suppress NRF2 activity
8. Mechanical stress & cell damage	Eye rubbing (frequency) [39, 73, 87]	Increased	Repetitive mechanical stress triggers inflammation and MMP activation; a major risk factor
	Keratocyte apoptosis [87, 97]	Increased	Enhanced cell death contributes to stromal thinning and weakening
9. Micronutrients & antioxidants	Vitamin D [177]	Decreased	Deficiency reduces antioxidant protection and impairs the corneal barrier
	Vitamin C (serum) [177]	Increased	Likely a compensatory systemic response to oxidative stress
	Vitamin C (tear levels) [177]	Unchanged	Local levels remain stable despite systemic changes
	Vitamin B12 [177]	Unchanged	No significant difference observed
	Selenium, Copper, Zinc [127]	Decreased	Deficiencies in these cofactors may exacerbate oxidative damage
	Lactoferrin (iron-binding protein) [132, 177]	Decreased	Lower levels compromise tear film stability and anti-inflammatory function
10. Tear Film & ocular surface changes	Tear secretion [96]	Decreased	Reduced production destabilizes the tear film, worsening dry eye
	Ocular surface temperature [200]	Increased	Elevated due to inflammation and mechanical stress, accelerating evaporation
	Tear protein levels [105]	Decreased	Lower overall protein content compromises tear quality
	Tear proteolytic activity [105]	Increased	Excess proteases degrade essential tear film components
11. Clinical observations & contact lens use	TBUT [40]	Decreased	Shorter TBUT indicates poor tear film stability
	Goblet cell count [40]	Decreased	Reduced mucin secretion destabilizes the tear film
	External ocular symptoms [40]	Increased	Heightened discomfort and irritation

*CAST* = calpastatin; *CCL5* = C–C motif chemokine ligand 5; *COL4A3* = collagen type IV alpha 3 chain; *COL4A4* = collagen type IV alpha 4 chain; *COL5A1* = collagen type V alpha 1 chain; *DOCK9* = dedicator of cytokinesis 9; *ECM* = extracellular matrix; *eIF/mTOR* = eukaryotic initiation factor/mammalian target of rapamycin; *FNDC3B* = fibronectin type III domain containing 3B; *FOXO1* = forkhead box O1; *HGF* = hepatocyte growth factor; *IFN-γ* = interferon-gamma; IL = interleukin; *IL-1α* = interleukin 1-alpha; *IL-1β* = interleukin-1 beta; *IL-1RN* = interleukin-1 receptor antagonist; *KEAP1* = kelch-like ech-associated protein 1; *LOX* = lysyl oxidase; *MMP* = matrix metalloproteinase; *NGF* = nerve growth factor; *NRF2* = nuclear factor erythroid 2-related factor 2; *RAB3-GAP1* = RAB3 GTPase activating protein 1; *SLC4A11* = solute carrier family 4 member 11; *SOD1* = superoxide dismutase 1; *TBUT* = tear break-up time; *TGF-β* = transforming growth factor beta; *TGFBI* = transforming growth factor beta induced; *Th1* = T helper 1; *TIMP* = tissue inhibitor of metalloproteinases; *TNF-α* = tumor necrosis factor-alpha; *VEGF* = vascular endothelial growth factor; *VSX1* = visual system homeobox 1; *ZEB1* = zinc finger e-box binding homeobox 1; *ZNF469* = zinc finger protein 469

#### Inflammation

Multiple studies suggest that inflammatory cytokines involved in OSDs also contribute to KC pathogenesis [36, 105, 179, 201]. Elevated tear levels of interleukins (IL-1β, IL-5, IL-6, IL-8], transforming growth factor-beta (TGF-β), C–C motif ligand 5 (CCL5), and cytokines associated with T helper (Th)-1 [IL-12, tumor necrosis factor (TNF)-α, IFN-γ], Th2 (IL-4, IL-10, IL-13), and Th17 (IL-17) pathways have been reported in KC patients [14, 67, 111, 123, 130, 209, 215]. Many of these, particularly IL-6, IL-17A, TNF-α, IL-8, and matrix metalloproteinase (MMP)-9, are also elevated in DED [170, 190, 199, 210].

A meta-analysis of seven studies (n = 374 eyes) confirmed significantly increased tear IL-1β, IL-6, and TNF-α levels in KC but found no difference in IL-4 or IL-10, reflecting inconsistent Th2 involvement [171].

Contrarily, Jun et al. observed decreased IL-12, TNF-α, CCL5, IL-4, and IL-13 —cytokines typically associated with Th1 and Th2 responses— while IL-17 remained elevated [62]. This discrepancy highlights immune activation variability across KC populations, possibly due to methodological differences, sample heterogeneity, or disease stage. Among these, IL-17 stands out as a consistently elevated and mechanistically important cytokine, known to induce fibroblast activation and upregulate MMP-9, thereby contributing to inflammatory and autoimmune diseases, such as Sjogren’s syndrome, as well as KC and DED [43, 60, 62]. Jun et al. attributed the reduced levels of other cytokines to localized inflammation despite systemic elevations in IL-1, IL-6, and TNF-α being reported in KC patients [62, 117, 124].

These cytokines and inflammatory mediators can trigger extracellular matrix (ECM) degradation by inducing oxidative stress and disrupting the protease-antiprotease balance for example, by upregulating MMPs [99, 101, 171, 199]. This cascade drives hallmark features of KC, including stromal thinning, Bowman’s layer disruption, and keratocyte apoptosis [97, 125]. Supporting this theory, studies have revealed correlations between changes in specific immuno-inflammatory parameters with corneal ectatic features, keratometry changes, and pachymetry measurements [14, 67, 102, 121, 130]. Wilson et al. suggested KC keratocytes that are hypersensitive to IL-1, predisposing them to apoptosis—a finding consistent with decreased stromal keratocyte density [18, 138].

In summary, while cytokine profiles vary across studies, the Th17-dominated immune response—especially persistent IL-17 elevation—alongside MMP-driven ECM remodeling, represents a core inflammatory mechanism in KC progression.

#### MMP cascades

MMPs are zinc-dependent enzymes produced as inactive precursors by corneal epithelial and stromal cells, later activated by other proteases [174]. MMP-9 plays a key role in ECM remodeling [138, 174] and is upregulated by pro-inflammatory mediators such as TNF-α, IL-1β, TGF-β, and Nuclear factor kappa light chain enhancer of activated B cells (NF-κB), with contribution from mitogen-activated protein kinase (MAPK) and activator protein 1 (AP1). Its activity is regulated by tissue inhibitors of metalloproteinases (TIMPs) and can be suppressed by agents such as corticosteroids, cyclosporine A (CsA), vitamin C, and modulation of extracellular signal-regulated kinase 1/2 (ERK1/2) [153]. IL-6, IL-8, and platelet-derived growth factor (PDGF) have also been implicated in regulating MMP cascades [97].

In physiological conditions, the MMP-TIMP balance preserves ECM homeostasis. Disruption—especially via inflammation or lacrimal dysfunction—can lead to MMP overactivity [138, 186].

MMP overexpression is implicated in ocular diseases such as DED and KC. In DED, tear MMP-9 is elevated, correlating with increased osmolarity, tight junction disruption, and inflammation; however, its increase appears predominantly in advanced stages, limiting its utility as a screening tool [45, 174, 186]. On the other hand, a recent study has shown that MMP-9 can be a screening marker for patients at risk of KC as its overexpression has been observed in some patients with subclinical disease. In addition, MMP-9 can be used to determine the severity of KC since an association has been observed between its levels and KC severity [138].

Further, MMPs (particularly MMP-1, -3, -9, and -13) positively correlate with tear osmolarity and negatively with Schirmer’s test and tear break-up time (TBUT) [174]. Furthermore, another study showed that MMP-9 inhibition improved signs and symptoms of OSDs [153]. In KC, increased MMP-1, -3, -7, -9, and -13 contribute to stromal thinning through ECM degradation, whereas MMP-2, derived from stromal cells, showed inconsistent associations with collagen remodeling [42, 97, 138]. Notably, tear MMP-9 levels appeared to track with KC severity [101, 138].

Evidence consistently implicates MMPs—especially MMP-9—as central players in both KC and DED. While the causal direction remains debatable, their diagnostic and therapeutic relevance are clear. The variability in MMP-2 findings warrants further clarification, but the convergence of MMP dysregulation across OSDs makes them valuable biomarkers and targets for intervention.

#### LOX

Lysyl oxidase (LOX), a copper-dependent enzyme, stabilizes the ECM through lysine cross-linking in collagen and elastin [97]. KC is associated with LOX gene polymorphisms, and reduced LOX expression and activity have been observed [97]. One study linked LOX overexpression in vascular smooth muscle cells to oxidative stress—a key player in both KC and DED [84, 122, 181]. LOX dysregulation may, therefore, exacerbate ECM degradation in the cornea and conjunctiva in KC and DED via oxidative pathways [162].

While evidence supports a mechanistic role for LOX deficiency in KC progression, its dual involvement in oxidative stress pathways adds complexity. These findings position LOX not only as a structural modulator but also as a potential therapeutic node in both KC and DED.

#### Sex hormones

Sex hormone involvement in KC remains ambiguous. While Fink et al. found no sex-based differences in KC progression [55], other studies associate disease onset with hormonal changes during puberty [177] and progression during pregnancy, likely via estrogen-mediated modulation of corneal and MMP activity. Similar discrepancies appear in DED research—some Link postmenopausal status to disease, while others report no impact of 17β-estradiol despite associations with meibomian dysfunction and elevated MMP-2 and -9 [86, 115, 120, 193].

Hormone replacement therapy has also been associated with worsening dry eye symptoms [24]. Additionally, altered expression of sex hormone receptors—specifically increased androgen and estrogen receptors and decreased progesterone receptors—has been reported in KC corneal epithelium [177].

Changes in luteinizing hormone (LH)/follicle-stimulating hormone (FSH) ratios have also been proposed as contributors to KC pathogenesis [177].

Despite numerous studies, hormonal contributions to KC and DED remain speculative due to inconclusive evidence. The interplay between estrogen, MMP activity, and glandular function deserves further mechanistic investigation before hormonal modulation can be considered clinically relevant.

#### VEGF and NGF

Vascular endothelial growth factor (VEGF) affects inflammation and vascular dynamics and may link KC with DED. The cornea’s dense innervation is regulated by nerve growth factor (NGF), which is elevated in KC [105]. However, VEGF findings are mixed—some studies report reduced or unchanged levels in KC corneas [22, 58]. VEGF may be driven by NGF and promote MMP-9 via ERK activation, suggesting a possible pathogenic mechanism in KC [174].

DED studies also show variable VEGF data—some indicate elevated tear VEGF [156, 188], and others have no serum difference [46]. VEGF may exacerbate DED by increasing vascular permeability and modulating immune cell infiltration, and inflammatory cytokines in DED may further enhance VEGF production, creating a feedback loop that sustains ocular surface inflammation [156, 188, 211].

The VEGF-NGF axis is a promising but underexplored area in KC and DED. While associations with MMP-9 and inflammation suggest pathogenic relevance, the inconsistent findings and lack of longitudinal data limit its current translational utility.

#### Genetics

Genetic factors significantly contribute to the development of KC. Genome-wide association studies (GWAS), candidate gene sequencing, whole-exome sequencing (WES), and candidate gene association studies have identified numerous loci associated with KC [167]. A positive family history also increases the risk of KC [98, 100]. However, the interpretation of genetic contributions is complicated by small variant effect sizes, population heterogeneity, and limited knowledge of functional mechanisms [167].

Identified single nucleotide polymorphisms (SNPs) involve genes related to ECM integrity [e.g., LOX, collagen types IV and V, fibronectin type III domain containing 3B (FNDC3B)], proteolysis [e.g., calpastatin (CAST)], cellular transport [e.g., solute carrier family 4 member 11 (SLC4A11)], inflammation (e.g., IL-1α, IL-1β, IL-1RN), and gene expression regulation [e.g., microRNA184, hepatocyte growth factor (HGF), superoxide dismutase 1 (SOD1)]. Transcription factors such as zinc finger protein 469 (ZNF469), zinc finger E-box binding homeobox 1 (ZEB1), visual system homeobox 1 (VSX1), and forkhead box O1 (FOXO1) are also implicated [59, 108, 167].

DED and KC exhibit genetic susceptibility that may involve similar pathways, particularly those associated with inflammation, ECM integrity, and oxidative stress.

DED and KC share overlapping genetic pathways involving inflammation, ECM stability, and oxidative stress. TNF-α, IL-1 family genes (IL-1α, IL-1β, and IL-1RN), and antioxidant genes such as SOD1 are common to both collagen type V alpha 1 chain (COL5A1) has been linked specifically to KC, while mucin genes such as MUC1, MUC5AC, and MUC5B are relevant to DED [61, 93, 131, 147, 212].

Although strong evidence supports a genetic basis for KC and overlapping pathways with DED, inconsistencies remain due to limited functional validation. Future work must clarify gene-environment interactions, particularly in diverse populations, and prioritize functionally impactful variants to translate genetic insights into clinical utility.

#### Transcriptomic and proteomic studies

##### CRF GWAS loci with GTEx e/sQTLs colocalization

Jiang et al. investigated genetic influences on corneal biomechanical properties by colocalizing 181 GWAS loci for corneal resistance factor (CRF) with genotype-tissue expression (GTEx) expression quantitative trait loci (eQTLs) and splicing quantitative trait loci (sQTLs). Approximately 26.5% of CRF-associated loci colocalized with GTEx-derived expression or splicing QTLs, identifying genes with high, tissue-specific expression in corneal stromal cells. These genes are involved in signaling, mechano-sensing, and tensile strength, providing insight into KC pathogenesis. Notably, some loci did not colocalize, suggesting limbal stromal responsiveness to context-specific cues beyond the scope of GTEx datasets [196].

##### eIF and mTOR

Shinde et al. used mass spectrometry on KC corneas and found upregulation of eukaryotic initiation factor (eIF)-2/eIF-4 and mammalian target of rapamycin (mTOR) pathways, involving translation, RNA metabolism, and protein degradation. Proteins related to vesicle transport and cytoskeletal structure were also elevated. Conversely, ECM proteins, acute-phase reactants, and classical complement components were downregulated, suggesting ER stress and impaired integrated stress response (ISR) [146].

##### Wnt signaling

Transcriptomic analyses have shifted the focus from KC as a pure stromal disorder to one involving epithelial dysregulation. RNA sequencing and immunohistochemistry reveal the downregulation of Wingless-related integration site (Wnt), Hedgehog, and Notch1 signaling pathways in the KC epithelium—key regulators of differentiation and intercellular signaling [135, 165]. Neurogenic locus notch homolog protein 1 (Notch1) and plasmolipin expression were significantly reduced, indicating impaired epithelial cell differentiation [135]. Reduced Wnt10A levels correlated with disease severity and may contribute to decreased collagen type I synthesis and disrupted epithelial-stromal signaling, thereby weakening corneal architecture [165].

##### NRF2

Under physiological conditions, the cornea counters reactive oxygen species (ROS) through antioxidant systems regulated by nuclear factor erythroid 2-related factor 2 (NRF2). In homeostasis, NRF2 remains cytoplasmically sequestered by Kelch-like ECH-associated protein 1 (KEAP1), which facilitates its ubiquitination via the Cullin 3 (CUL3) complex [64, 79]. Under oxidative stress, ROS triggers conformational changes in the KEAP1-NRF2 complex, resulting in NRF2 stabilization and nuclear translocation, where it activates antioxidant genes [159].

In KC, this protective mechanism appears compromised. Proteomic data showed reduced levels of NRF2-regulated proteins, including antioxidant enzymes and complement pathway components. Transcriptomic studies further revealed the downregulation of NRF2 target genes, such as heme oxygenase 1 (HMOX1), which mediates iron homeostasis and acute-phase responses. Immunofluorescence confirms decreased NRF2 and increased KEAP1 expression in KC corneas, suggesting that defective oxidative stress resolution contributes to disease progression [146, 159].

Transcriptomic and proteomic studies reveal that KC involves not only stromal alterations but also significant dysregulation of epithelial and molecular processes. Key findings include disrupted protein synthesis (eIF/mTOR), impaired oxidative stress response (NRF2), and downregulated epithelial signaling pathways (Wnt, Notch1). Colocalization analyses partially link genetic variants to corneal tissue function, though tissue-specific gaps remain.

These insights suggest that KC pathogenesis extends beyond collagen breakdown, involving stress response failure and impaired epithelial-stromal crosstalk. Future therapies may benefit from targeting these molecular pathways rather than focusing solely on biomechanical reinforcement.

#### Microtrauma

Frequent eye rubbing is common in OSDs such as DED, MGD, blepharitis, and AKC [141]. It mechanically disrupts the ocular surface, reduces tear stability and secretion, and triggers histamine release, promoting a cycle of itching, rubbing, and inflammation [107, 144, 194].

In KC, rubbing is proposed to act as a friction-induced disease (FRD), where mechanical stress and inflammation weaken the corneal structure and shear strength, contributing to ectasia [51, 172]. Other mechanisms include localized heat from eyelid closure, vascular proximity of the palpebral conjunctiva, and inflammation-induced remodeling [71]. Despite this, controlled thermal therapy can benefit MGD [94, 137]. Rubbing also elevates intraocular pressure (IOP), potentially impacting corneal integrity [63, 109]. Chronic, compulsive rubbing has even been identified as an independent KC risk factor [87].

Microscopically, rubbing disturbs the keratocyte apoptosis–proliferation balance [87],

while immunologically, it induces cytokine (e.g., IL-6, TNF-α) release and upregulates MMPs (MMP-1, -9, and -13), enhancing ECM degradation (e.g., IL-6, TNF-α) [39, 73, 87, 148].

The evidence positions eye rubbing as a key behavioral trigger linking mechanical injury with inflammation and proteolysis in KC and DED. Its recognition as a modifiable risk factor underscores the importance of preventive counseling for individuals at risk.

#### Micronutrients

Micronutrients, including vitamins and trace elements, may play parallel roles in the pathogenesis of KC and DED, as recent studies suggest shared deficiencies and dysfunctions [177].

#### Vitamin D

Vitamin D deficiency is implicated in both diseases. In KC, it supports antioxidant defense and corneal endothelial integrity. In DED, it preserves epithelial barrier function and enhances tear secretion by modulating calcium absorption [158]. Vitamin D supplementation has shown therapeutic benefits in DED [106, 134].

#### Vitamin C

Vitamin C facilitates collagen synthesis and tissue repair. In KC, systemic levels may rise compensatorily, though tear levels remain unchanged [177]. Supplementation improves ocular surface status by reducing oxidative damage and stabilizing the tear film [95, 110, 152].

#### Vitamin B12

Vitamin B12 serum levels appear similar in KC patients and controls, but they may still influence the disease via effects on collagen-synthesizing enzymes. [177]. In DED, deficiency is linked to neuropathic symptoms and ocular pain. Supplementation alleviates severe DED symptoms [158].

#### Trace elements

Selenium, zinc, copper, and iron are essential cofactors for antioxidant enzymes and ECM-related processes [177]. Advanced KC is associated with lower serum selenium, copper, and zinc [127]. Reduced tear pH may impair copper transport to the central cornea [97], and decreased tear lactoferrin suggests micronutrient dysregulation—especially of vitamins D, C, B12, and trace elements—represents a shared biochemical vulnerability in KC and DED, affecting antioxidant capacity, ECM integrity, and neurosensory health. Their diagnostic and therapeutic potential merit further exploration [177].

### Suggested mechanisms for KC’s contribution to OSD pathogenesis

#### Temperature of the tear film microenvironment

KC patients often demonstrate reduced tear secretion, elevated ocular surface disease index (OSDI) scores, and altered thermal sensitivity, suggesting dysfunctional corneal thermoregulation [96]. Rubbing may further increase corneal temperature via mechanical stress and inflammatory activation [200].

#### Tear film microenvironment components

KC alters tear quality and distribution over the irregular cornea, potentially triggering or worsening OSDs [5, 57, 140]. Total tear protein levels decline while proteolytic activity and transporter/glycoprotein levels increase [105].

Lactoferrin, an iron-chelating antioxidant glycoprotein critical for reducing oxidative stress and inflammation, is lowered in DED and MGD and linked to KC via its immunomodulatory role, inhibiting cytokines such as IL-1, IL-2, IL-6, and TNF-α [56, 97, 132, 202]. Furthermore, its therapeutic potential in DED and KC has been recently proposed [202].

#### Lipid layer thickness and blink rate

MGD in KC reduces lipid layer thickness, increasing tear evaporation and epithelial vulnerability [30, 142, 175, 187, 208, 214]. Blinking, especially partial blinks, distributes tears and maintains lipid integrity [65, 119, 164, 189]. In mild KC, glandular compensation may preserve lipid integrity; however, a negative correlation exists between corneal curvature and partial blink rate [142] indicates that increasing disease severity exacerbates tear film instability and ocular surface stress [157, 187].

#### Corneal epithelium

KC induces distinct epithelial changes that may disrupt ocular surface stability. Typically, epithelial thinning occurs at the cone’s apex, accompanied by thickening around it—a donut-like pattern that compensates for stromal irregularities [53]. However, this pattern varies; some report non-uniform thickening/thinning [68]. Higher peripheral epithelial cell density and reduced apex density suggest mechanical stress and increased apoptosis risk [145, 178]. Impaired epithelial-stromal interaction further weakens corneal integrity and influences responses to treatments such as CXL and contact lenses. While one study observed increased epithelial thickness in DED [89], several others found superior corneal thinning correlating with disease severity [85, 198, 216].

Orbscan (Orbtek, Inc., Salt Lake City, UT) data confirm reduced central and mid-peripheral thickness in DED, attributed to chronic desiccation, immune activation, and tear film reduction [33].

#### Corneal sub-basal plexus nerves

The corneal sub-basal nerve plexus—critical for blinking, tear secretion, and homeostasis—is compromised in KC, showing reduced density and disorganization [75, 91]. This results in diminished sensation and delayed healing [27, 113]. While contact lens use may contribute, recent evidence suggests hypoesthesia is intrinsic to KC [82, 96, 160], supported by lower sensitivity in KC lens users compared to controls [82]. Moreover, corneal hypoesthesia in KC and subclinical KC cases has been associated with abnormal cellular changes, decreased tear production, and a lack of goblet cells [75]. The accelerated death of basal epithelial cells, potentially triggered by the release of intracellular proteolytic enzymes, has been proposed as a mechanism underlying the reduced corneal sensitivity seen in KC patients [35, 37, 41, 52]. Severity of KC correlates with diminished corneal sensitivity [26, 75, 82].

In summary, KC disrupts the ocular surface microenvironment far beyond corneal shape changes. Tear film instability, altered lipid layers, and abnormal blink patterns indicate a failing compensatory system that worsens OSD progression. The epithelial remodeling reflects localized mechanical stress that may undermine treatment outcomes. Nerve plexus alterations reveal a neuro-inflammatory dimension often overlooked. Together, these insights argue for a holistic KC management approach targeting tear film, epithelial integrity, and neural health—not just stromal biomechanics.

#### Ocular surface microbiome alterations

Emerging evidence suggests that microbiome imbalance may contribute to KC pathogenesis. Although findings are inconsistent, several studies report distinct microbial profiles in KC patients. Tunç et al. proposed that dysbiosis could trigger Toll-like receptor-mediated inflammation [169], aligning with broader theories of ocular surface immune activation.

Specific microbial shifts have been noted: *Pelomonas* and *Ralstonia* were exclusive to untreated KC [203], *Aquabacterium* predominated in KC, while *Shigella* dominated controls [206]. Another study found increased *Alphaproteobacteria* and reduced *Actinobacteria* and *Firmicutes-Bacilli* in KC, consistent with dysbiosis [217]. However, Kaur et al., using deep metagenomic sequencing, found no significant differences between KC and healthy corneas [213], challenging the idea of a consistent microbial signature.

These findings suggest that while a definitive microbiome profile in KC remains elusive, dysbiosis may contribute to ocular surface inflammation. Clinically, this supports the view that KC may overlap with or represent a subclinical form of OSD involving barrier disruption, tear film instability, and microbial imbalance. Whether these changes precede or follow stromal remodeling remains unclear. Incorporating microbiome assessment into KC evaluation may provide diagnostic and therapeutic insights. Longitudinal studies are needed to establish causality and guide interventions.

### Therapeutic interventions in KC: dual impact on the OSD

**Fig. 3:**
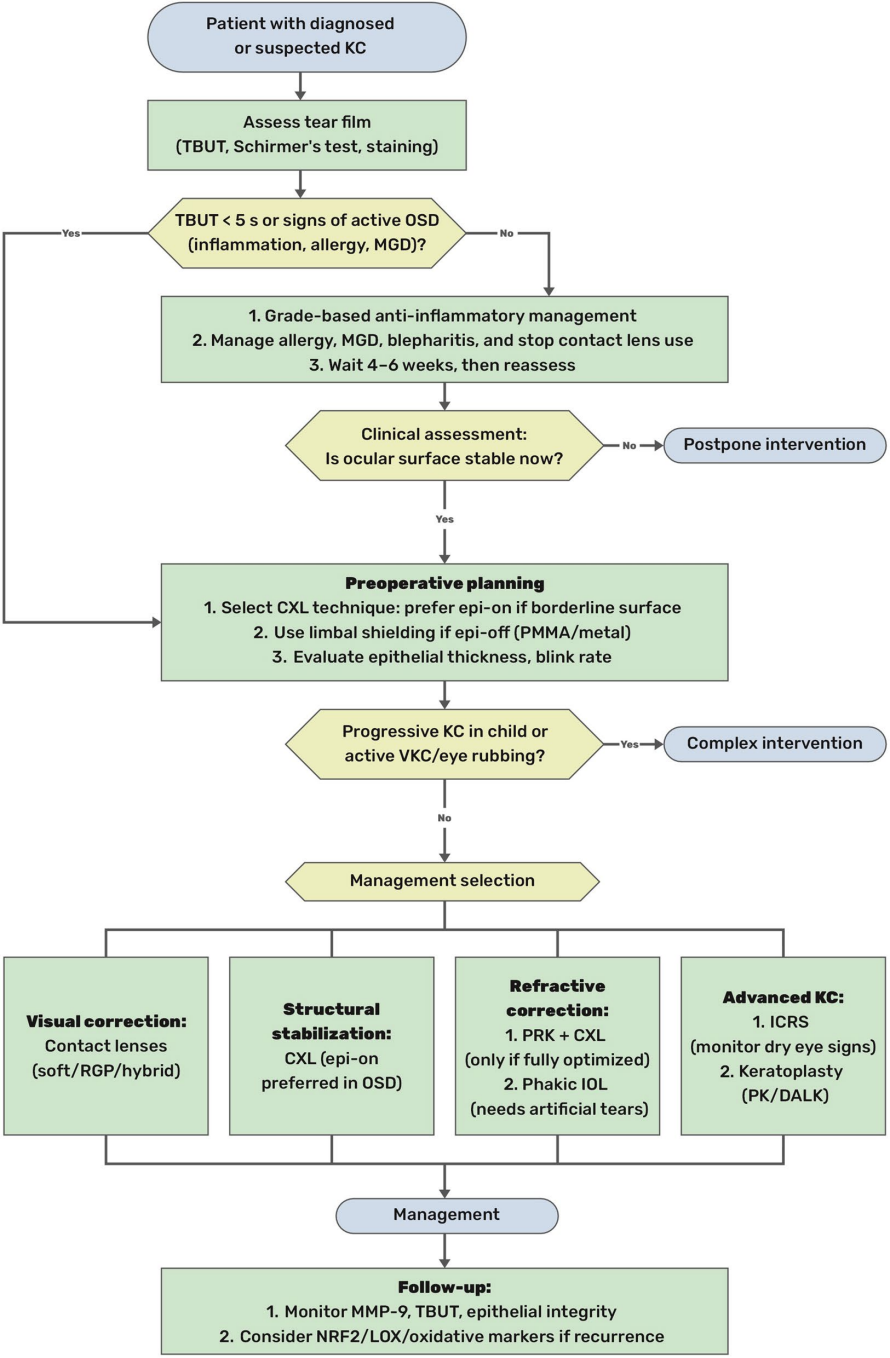
Clinical decision-making flowchart for keratoconus, factoring in the ocular surface microenvironment [5–13, 17, 19, 25, 32, 48, 50, 51, 53, 56, 68, 71, 74–78, 81, 83, 84, 90, 95, 97, 101, 104, 107, 110, 122, 133, 138, 142, 144–146, 151–154, 157, 159, 160, 162, 172, 177, 178, 181, 187, 191, 192, 194, 195, 202, 218–252]. CsA, cyclosporine A; CXL, corneal cross-linking; DALK, deep anterior lamellar keratoplasty; ICRS, intracorneal ring segment; IOL, intraocular lens; KC, keratoconus; LOX, lysyl oxidase; MGD, meibomian gland dysfunction; MMP-9, matrix metalloproteinase-9; NRF2, nuclear factor erythroid 2–related factor 2; OSD, ocular surface disease; PK, penetrating keratoplasty; PMMA, polymethylmethacrylate; PRK, photorefractive keratectomy; RGP, rigid gas permeable; TBUT, tear break-up time; VKC, vernal keratoconjunctivitis

Management strategies for KC vary by disease stage, including surgical and nonsurgical options [143, 173, 191]. However, coexisting or predisposing OSDs may impair treatment efficacy. Accurate visual and keratometric assessments can be compromised if OSD is not addressed beforehand [118]. Figure 3 illustrates a decision-making flowchart guiding KC management, considering the ocular surface.

#### Contact lenses

Contact lenses restore vision in about 75% of KC cases [151, 197], yet they can negatively impact the ocular surface. Their use decreases TBUT and goblet cell density—especially in atopic patients—and discomfort correlates with afferent nerve disruption [160] and possible lipid layer disturbance [28, 72, 183, 185]. Contact lens wear is uniquely associated with decreased keratocyte density in KC, suggesting epithelial damage from apoptotic cytokines, a feature absent in healthy eyes [25]. This supports the hypothesis that lens-induced microtrauma may worsen KC by upregulating MMP-9 and promoting inflammation [138].

Moreover, differential expression of intercellular adhesion molecule 1 (ICAM-1), vascular adhesion molecule 1 (VCAM-1), IL-6, and IL-10 has been observed in KC versus healthy contact lens wearers, further suggesting inflammation-linked progression [97, 105].

##### Contact lens-related disorders: GPC

Giant papillary conjunctivitis (GPC), an inflammatory condition mainly linked to contact lens wear [104], exacerbates KC and OSD by sustaining ocular surface inflammation [19], destabilizing the tear film, and damaging the epithelium. The irritation and itching provoke eye rubbing [17, 154], mechanically stressing the cornea and accelerating KC progression. In DED, GPC reduces blink efficiency and increases evaporation. Controlling GPC is essential to preserve tear stability and corneal integrity in KC and DED.

##### Contact lens-related disorders: LSCD

Prolonged contact lens wear may induce epithelial remodeling and compromise limbal stem cells (LSCs), essential for corneal epithelium regeneration [49, 80, 88, 89]. Limbal stem cell deficiency (LSCD) causes conjunctivalization and impairs corneal clarity and healing [195]. In KC, this amplifies existing epithelial deficits, scarring risk, and opacification. LSCD also exacerbates dry eye via inflammation and meibomian dysfunction [23], compounding OSD severity.

Section 4.2.1 highlights a clinical contradiction: while lenses are critical for vision in KC, they may worsen the ocular surface microenvironment. Their role in nerve disruption, epithelial damage, and inflammation suggests they are active disease modifiers. GPC and LSCD further support a need to rethink contact lens protocols through a KC-specific, microenvironment-aware approach.

#### CXL and ocular surface microenvironment

Despite newer KC treatments [126, 150, 182], CXL remains the primary method for halting progression, offering a minimally invasive approach [205]. It involves applying riboflavin (vitamin B2) and UVA exposure to generate oxygen radicals, cross-linking stromal collagen [205]. Effective stromal penetration of riboflavin is essential [29], though epithelial removal remains the main procedural obstacle [44, 47].

##### Impact of CXL on OSDs

CXL’s effect on OSDs is debated [92]. Some studies reported no significant changes in TBUT, Schirmer’s test, tear osmolarity, or staining three months post-procedure [116], while others reported temporary nerve loss but stable tear parameters by six months [78]. Earlier reviews lacked data on outcomes related to the ocular surface [103], though a recent review [168] identified one study where trans-epithelial CXL improved OSDI scores over epithelium-off (epi-off) CXL at one month [128, 192]. Transient denervation from epithelial removal may decrease blinking and increase evaporation, triggering short-term DED symptoms [48, 74, 78, 83]. Nonetheless, long-term improvements in DED have been reported with both epithelium-on (epi-on) and epi-off techniques [5]. Post-CXL, goblet cell density, and mucin layer integrity improve, enhancing tear stability [48, 192]. Iontophoretic CXL also yielded better vision, corneal flattening, and OSDI scores over two years [180].

##### Impact of OSDs on CXL

Conversely, OSDs may compromise CXL efficacy. Allergies and atopy have been associated with treatment failure and KC progression post-CXL [32, 50, 76, 81]. Children with KC and VKC show a 17.65% failure rate after accelerated CXL, with proteomic and genomic data supporting increased susceptibility [76].

Eye rubbing, partial LSCD (resulting from VKC), and chronic inflammation—exacerbated by UVA exposure—may deplete LSCs [66, 70]. Adult VKC is similarly linked to heightened inflammation, elevated androgen receptor expression [139], and greater postoperative keratitis risk from altered flora due to chronic topical steroid application for VKC management [31].

Molecular analyses reveal that CXL downregulates most MMPs (except MMP-2), thereby stabilizing the ECM. It also modulates prostaglandins (PGs), decreasing lumican and decorin while increasing mimecan and keratocan, reflecting improved collagen alignment [133].

Intraoperative limbal protection with metal or polymethylmethacrylate (PMMA) shield has preserved LSCs in vitro and cadaver eyes, supporting their use alongside steroids, mast cell stabilizers, and cyclosporine. [77, 90].

While transient ocular surface disruption post-CXL is well-documented, emerging evidence supports a reparative effect over time—especially with epi-on and iontophoretic techniques. However, the more clinically relevant concern is the bidirectional influence: OSDs, particularly VKC and LSCD, markedly reduce CXL efficacy. This appears to be less biomechanical and more a function of chronic inflammation and limbal niche exhaustion. These findings argue for proactive ocular surface management as a prerequisite to CXL, especially in younger and atopic patients.

### Limitations

Despite the comprehensive scope of this review, several limitations in the literature should be acknowledged. The included studies were notably heterogeneous in design, sample size, and methodologies used to assess ocular surface mediators. While many investigations have reported elevated inflammatory cytokines in KC patients, several studies did not report significant differences in certain mediators between normal eyes and those with KC. This inconsistency underscores inherent ambiguities in literature, possibly arising from variations in patient selection, analytical techniques, or temporal factors. Moreover, the number of cross-sectional studies limits our ability to draw causal inferences regarding the interplay between ocular surface dysfunction and KC progression. Confounding variables—such as environmental influences, genetic predispositions, and inter-observer variability—further complicate the interpretation of these findings. Future research should focus on employing standardized, longitudinal methodologies to address these limitations.

### Conclusion

KC is not solely a biomechanical disorder, but a complex condition shaped by ocular surface inflammation, oxidative stress, epithelial dysfunction, and tear film instability. Its overlap with OSDs suggests a bidirectional pathophysiology that fuels progression and undermines treatment efficacy.

Effective management requires a shift toward microenvironment-centered evaluation, integrating inflammatory, neural, and epithelial markers with conventional diagnostics. Addressing coexisting OSDs is essential to optimize outcomes, especially in contact lens use and cross-linking.

Future studies should adopt multiomic, longitudinal approaches to clarify mechanisms and guide targeted, preventive strategies.

## Supplementary Information

Additional file 1.

## Data Availability

The data that support the findings of this study are available from the corresponding author upon reasonable request.

## Abbreviations

AKC: Atopic keratoconjunctivitis
CD: Cluster of differentiation
CD-11b: Cluster of differentiation11b
CsA: Cyclosporine A
CXL: Corneal cross-linking
DALK: Deep anterior lamellar keratoplasty
DC: Dendritic cell
ECM: Extracellular matrix
ECP: Eosinophil cationic protein
Foxp3: Forkhead box P3
ICRS: Intracorneal ring segment
IFN: Interferon
IL: Interleukin
ILC: Innate lymphoid cells
IVCM: In vivo confocal microscopy
LC: Lamina cribrosa
LSCD: Limbal stem cell deficiency
MC: Mast cells
MCP: Monocyte chemoattractant protein
MIP: Major intrinsic polypeptide
MMP: Matrix metalloproteinase
NGF: Nerve growth factor
NITBUT: Noninvasive tear break-up time
NKC: Natural killer cells
NSAID: Nonsteroidal anti-inflammatory drugs
OCT: Optical coherence tomography
OSDI: Ocular surface disease index
PK: Penetrating keratoplasty
RANTES: Regulated on activation, normal T-cell expressed and secreted
TGF: Transforming growth factor
Th: T helper
TNF: Tumor necrosis factor
VKC: Vernal keratoconjunctivitis
