# Supplementary material for: The microenvironment of ocular surface in keratoconus: a systematic review
Source: Eye Vis (Lond). 2025 Sep 24;12:37. doi: 10.1186/s40662-025-00454-0 (PMC12459042; doi:10.1186/s40662-025-00454-0)
Supplement: Supplementary file 1 — Additional file 1. [file 40662_2025_454_MOESM1_ESM.docx]

# Quality Assessment of Controlled Intervention Studies

- Study Quality Assessment Tools

| **Criteria** | **Yes** | **No** | **Other (CD, NR, NA)*** |
| --- | --- | --- | --- |
| 1. Was the study described as randomized, a randomized trial, a randomized clinical trial, or an RCT? |  |  |  |
| 2. Was the method of randomization adequate (i.e., use of randomly generated assignment)? |  |  |  |
| 3. Was the treatment allocation concealed (so that assignments could not be predicted)? |  |  |  |
| 4. Were study participants and providers blinded to treatment group assignment? |  |  |  |
| 5. Were the people assessing the outcomes blinded to the participants' group assignments? |  |  |  |
| 6. Were the groups similar at baseline on important characteristics that could affect outcomes (e.g., demographics, risk factors, co-morbid conditions)? |  |  |  |
| 7. Was the overall drop-out rate from the study at endpoint 20% or lower of the number allocated to treatment? |  |  |  |
| 8. Was the differential drop-out rate (between treatment groups) at endpoint 15 percentage points or lower? |  |  |  |
| 9. Was there high adherence to the intervention protocols for each treatment group? |  |  |  |
| 10. Were other interventions avoided or similar in the groups (e.g., similar background treatments)? |  |  |  |
| 11. Were outcomes assessed using valid and reliable measures, implemented consistently across all study participants? |  |  |  |
| 12. Did the authors report that the sample size was sufficiently large to be able to detect a difference in the main outcome between groups with at least 80% power? |  |  |  |
| 13. Were outcomes reported or subgroups analyzed prespecified (i.e., identified before analyses were conducted)? |  |  |  |
| 14. Were all randomized participants analyzed in the group to which they were originally assigned, i.e., did they use an intention-to-treat analysis? |  |  |  |

| **Quality Rating (Good, Fair, or Poor)** |
| --- |
| Rater #1 initials: |
| Rater #2 initials: |
| Additional Comments (If POOR, please state why): |

*CD, cannot determine; NA, not applicable; NR, not reported

**Guidance for Assessing the Quality of Controlled Intervention Studies**

The guidance document below is organized by question number from the tool for quality assessment of controlled intervention studies.

**Question 1. Described as randomized**

Was the study described as randomized? A study does not satisfy quality criteria as randomized simply because the authors call it randomized; however, it is a first step in determining if a study is randomized

**Questions 2 and 3. Treatment allocation–two interrelated pieces**

Adequate randomization: Randomization is adequate if it occurred according to the play of chance (e.g., computer generated sequence in more recent studies, or random number table in older studies).
Inadequate randomization: Randomization is inadequate if there is a preset plan (e.g., alternation where every other subject is assigned to treatment arm or another method of allocation is used, such as time or day of hospital admission or clinic visit, ZIP Code, phone number, etc.). In fact, this is not randomization at all–it is another method of assignment to groups. If assignment is not by the play of chance, then the answer to this question is no.
There may be some tricky scenarios that will need to be read carefully and considered for the role of chance in assignment. For example, randomization may occur at the site level, where all individuals at a particular site are assigned to receive treatment or no treatment. This scenario is used for group-randomized trials, which can be truly randomized, but often are "quasi-experimental" studies with comparison groups rather than true control groups. (Few, if any, group-randomized trials are anticipated for this evidence review.)

Allocation concealment: This means that one does not know in advance, or cannot guess accurately, to what group the next person eligible for randomization will be assigned. Methods include sequentially numbered opaque sealed envelopes, numbered or coded containers, central randomization by a coordinating center, computer-generated randomization that is not revealed ahead of time, etc.

**Questions 4 and 5. Blinding**

Blinding means that one does not know to which group–intervention or control–the participant is assigned. It is also sometimes called "masking." The reviewer assessed whether each of the following was blinded to knowledge of treatment assignment: (1) the person assessing the primary outcome(s) for the study (e.g., taking the measurements such as blood pressure, examining health records for events such as myocardial infarction, reviewing and interpreting test results such as x ray or cardiac catheterization findings); (2) the person receiving the intervention (e.g., the patient or other study participant); and (3) the person providing the intervention (e.g., the physician, nurse, pharmacist, dietitian, or behavioral interventionist).

Generally placebo-controlled medication studies are blinded to patient, provider, and outcome assessors; behavioral, lifestyle, and surgical studies are examples of studies that are frequently blinded only to the outcome assessors because blinding of the persons providing and receiving the interventions is difficult in these situations. Sometimes the individual providing the intervention is the same person performing the outcome assessment. This was noted when it occurred.

**Question 6. Similarity of groups at baseline**

This question relates to whether the intervention and control groups have similar baseline characteristics on average especially those characteristics that may affect the intervention or outcomes. The point of randomized trials is to create groups that are as similar as possible except for the intervention(s) being studied in order to compare the effects of the interventions between groups. When reviewers abstracted baseline characteristics, they noted when there was a significant difference between groups. Baseline characteristics for intervention groups are usually presented in a table in the article (often Table 1).

Groups can differ at baseline without raising red flags if: (1) the differences would not be expected to have any bearing on the interventions and outcomes; or (2) the differences are not statistically significant. When concerned about baseline difference in groups, reviewers recorded them in the comments section and considered them in their overall determination of the study quality.

**Questions 7 and 8. Dropout**

"Dropouts" in a clinical trial are individuals for whom there are no end point measurements, often because they dropped out of the study and were lost to follow up.

Generally, an acceptable overall dropout rate is considered 20% or less of participants who were randomized or allocated into each group. An acceptable differential dropout rate is an absolute difference between groups of 15% points at most (calculated by subtracting the dropout rate of one group minus the dropout rate of the other group). However, these are general rates. Lower overall dropout rates are expected in shorter studies, whereas higher overall dropout rates may be acceptable for studies of longer duration. For example, a 6-month study of weight loss interventions should be expected to have nearly 100% follow-up (almost no dropouts–nearly everybody gets their weight measured regardless of whether or not they actually received the intervention), whereas a 10-year study testing the effects of intensive blood pressure lowering on heart attacks may be acceptable if there is a 20% to 25% dropout rate, especially if the dropout rate between groups was similar. The panels for the NHLBI systematic reviews may set different levels of dropout caps.

Conversely, differential dropout rates are not flexible; there should be a 15% cap. If there is a differential dropout rate of 15% or higher between arms, then there is a serious potential for bias. This constitutes a fatal flaw, resulting in a poor quality rating for the study.

**Question 9. Adherence**

Did participants in each treatment group adhere to the protocols for assigned interventions? For example, if Group 1 was assigned to 10 mg/day of Drug A, did most of them take 10 mg/day of Drug A? Another example is a study evaluating the difference between a 30-pound weight loss and a 10-pound weight loss on specific clinical outcomes (e.g., heart attacks), but the 30-pound weight loss group did not achieve its intended weight loss target (e.g., the group only lost 14 pounds on average). A third example is whether a large percentage of participants assigned to one group "crossed over" and got the intervention provided to the other group. A final example is when one group that was assigned to receive a particular drug at a particular dose had a large percentage of participants who did not end up taking the drug or the dose as designed in the protocol.

**Question 10. Avoid other interventions**

Changes that occur in the study outcomes being assessed should be attributable to the interventions being compared in the study. If study participants receive interventions that are not part of the study protocol and could affect the outcomes being assessed, and they receive these interventions differentially, then there is cause for concern because these interventions could bias results. The following scenario is another example of how bias can occur. In a study comparing two different dietary interventions on serum cholesterol, one group had a significantly higher percentage of participants taking statin drugs than the other group. In this situation, it would be impossible to know if a difference in outcome was due to the dietary intervention or the drugs.

**Question 11. Outcome measures assessment**

What tools or methods were used to measure the outcomes in the study? Were the tools and methods accurate and reliable–for example, have they been validated, or are they objective? This is important as it indicates the confidence you can have in the reported outcomes. Perhaps even more important is ascertaining that outcomes were assessed in the same manner within and between groups. One example of differing methods is self-report of dietary salt intake versus urine testing for sodium content (a more reliable and valid assessment method). Another example is using BP measurements taken by practitioners who use their usual methods versus using BP measurements done by individuals trained in a standard approach. Such an approach may include using the same instrument each time and taking an individual's BP multiple times. In each of these cases, the answer to this assessment question would be "no" for the former scenario and "yes" for the latter. In addition, a study in which an intervention group was seen more frequently than the control group, enabling more opportunities to report clinical events, would not be considered reliable and valid.

**Question 12. Power calculation**

Generally, a study's methods section will address the sample size needed to detect differences in primary outcomes. The current standard is at least 80% power to detect a clinically relevant difference in an outcome using a two-sided alpha of 0.05. Often, however, older studies will not report on power.

**Question 13. Prespecified outcomes**

Investigators should prespecify outcomes reported in a study for hypothesis testing–which is the reason for conducting an RCT. Without prespecified outcomes, the study may be reporting ad hoc analyses, simply looking for differences supporting desired findings. Investigators also should prespecify subgroups being examined. Most RCTs conduct numerous post hoc analyses as a way of exploring findings and generating additional hypotheses. The intent of this question is to give more weight to reports that are not simply exploratory in nature.

**Question 14. Intention-to-treat analysis**

Intention-to-treat (ITT) means everybody who was randomized is analyzed according to the original group to which they are assigned. This is an extremely important concept because conducting an ITT analysis preserves the whole reason for doing a randomized trial; that is, to compare groups that differ only in the intervention being tested. When the ITT philosophy is not followed, groups being compared may no longer be the same. In this situation, the study would likely be rated poor. However, if an investigator used another type of analysis that could be viewed as valid, this would be explained in the "other" box on the quality assessment form. Some researchers use a completers analysis (an analysis of only the participants who completed the intervention and the study), which introduces significant potential for bias. Characteristics of participants who do not complete the study are unlikely to be the same as those who do. The likely impact of participants withdrawing from a study treatment must be considered carefully. ITT analysis provides a more conservative (potentially less biased) estimate of effectiveness.

**General Guidance for Determining the Overall Quality Rating of Controlled Intervention Studies**

The questions on the assessment tool were designed to help reviewers focus on the key concepts for evaluating a study's internal validity. They are not intended to create a list that is simply tallied up to arrive at a summary judgment of quality.

Internal validity is the extent to which the results (effects) reported in a study can truly be attributed to the intervention being evaluated and not to flaws in the design or conduct of the study–in other words, the ability for the study to make causal conclusions about the effects of the intervention being tested. Such flaws can increase the risk of bias. Critical appraisal involves considering the risk of potential for allocation bias, measurement bias, or confounding (the mixture of exposures that one cannot tease out from each other). Examples of confounding include co-interventions, differences at baseline in patient characteristics, and other issues addressed in the questions above. High risk of bias translates to a rating of poor quality. Low risk of bias translates to a rating of good quality.

Fatal flaws: If a study has a "fatal flaw," then risk of bias is significant, and the study is of poor quality. Examples of fatal flaws in RCTs include high dropout rates, high differential dropout rates, no ITT analysis or other unsuitable statistical analysis (e.g., completers-only analysis).

Generally, when evaluating a study, one will not see a "fatal flaw;" however, one will find some risk of bias. During training, reviewers were instructed to look for the potential for bias in studies by focusing on the concepts underlying the questions in the tool. For any box checked "no," reviewers were told to ask: "What is the potential risk of bias that may be introduced by this flaw?" That is, does this factor cause one to doubt the results that were reported in the study?

NHLBI staff provided reviewers with background reading on critical appraisal, while emphasizing that the best approach to use is to think about the questions in the tool in determining the potential for bias in a study. The staff also emphasized that each study has specific nuances; therefore, reviewers should familiarize themselves with the key concepts.

# Quality Assessment of Systematic Reviews and Meta-Analyses

- Study Quality Assessment Tools

| **Criteria** | **Yes** | **No** | **Other (CD, NR, NA)*** |
| --- | --- | --- | --- |
| 1. Is the review based on a focused question that is adequately formulated and described? |  |  |  |
| 2. Were eligibility criteria for included and excluded studies predefined and specified? |  |  |  |
| 3. Did the literature search strategy use a comprehensive, systematic approach? |  |  |  |
| 4. Were titles, abstracts, and full-text articles dually and independently reviewed for inclusion and exclusion to minimize bias? |  |  |  |
| 5. Was the quality of each included study rated independently by two or more reviewers using a standard method to appraise its internal validity? |  |  |  |
| 6. Were the included studies listed along with important characteristics and results of each study? |  |  |  |
| 7. Was publication bias assessed? |  |  |  |
| 8. Was heterogeneity assessed? (This question applies only to meta-analyses.) |  |  |  |

| **Quality Rating (Good, Fair, or Poor)** |
| --- |
| Rater #1 initials: |
| Rater #2 initials: |
| Additional Comments (If POOR, please state why): |

*CD, cannot determine; NA, not applicable; NR, not reported

**Guidance for Quality Assessment Tool for Systematic Reviews and Meta-Analyses**

A systematic review is a study that attempts to answer a question by synthesizing the results of primary studies while using strategies to limit bias and random error. These strategies include a comprehensive search of all potentially relevant articles and the use of explicit, reproducible criteria in the selection of articles included in the review. Research designs and study characteristics are appraised, data are synthesized, and results are interpreted using a predefined systematic approach that adheres to evidence-based methodological principles.

Systematic reviews can be qualitative or quantitative. A qualitative systematic review summarizes the results of the primary studies but does not combine the results statistically. A quantitative systematic review, or meta-analysis, is a type of systematic review that employs statistical techniques to combine the results of the different studies into a single pooled estimate of effect, often given as an odds ratio. The guidance document below is organized by question number from the tool for quality assessment of systematic reviews and meta-analyses.

**Question 1. Focused question**

The review should be based on a question that is clearly stated and well-formulated. An example would be a question that uses the PICO (population, intervention, comparator, outcome) format, with all components clearly described.

**Question 2. Eligibility criteria**

The eligibility criteria used to determine whether studies were included or excluded should be clearly specified and predefined. It should be clear to the reader why studies were included or excluded.

**Question 3. Literature search**

The search strategy should employ a comprehensive, systematic approach in order to capture all of the evidence possible that pertains to the question of interest. At a minimum, a comprehensive review has the following attributes:

- Electronic searches were conducted using multiple scientific literature databases, such as MEDLINE, EMBASE, Cochrane Central Register of Controlled Trials, PsychLit, and others as appropriate for the subject matter.
- Manual searches of references found in articles and textbooks should supplement the electronic searches.

Additional search strategies that may be used to improve the yield include the following:

- Studies published in other countries
- Studies published in languages other than English
- Identification by experts in the field of studies and articles that may have been missed
- Search of grey literature, including technical reports and other papers from government agencies or scientific groups or committees; presentations and posters from scientific meetings, conference proceedings, unpublished manuscripts; and others. Searching the grey literature is important (whenever feasible) because sometimes only positive studies with significant findings are published in the peer-reviewed literature, which can bias the results of a review.

In their reviews, researchers described the literature search strategy clearly, and ascertained it could be reproducible by others with similar results.

**Question 4. Dual review for determining which studies to include and exclude**

Titles, abstracts, and full-text articles (when indicated) should be reviewed by two independent reviewers to determine which studies to include and exclude in the review. Reviewers resolved disagreements through discussion and consensus or with third parties. They clearly stated the review process, including methods for settling disagreements.

**Question 5. Quality appraisal for internal validity**

Each included study should be appraised for internal validity (study quality assessment) using a standardized approach for rating the quality of the individual studies. Ideally, this should be done by at least two independent reviewers appraised each study for internal validity. However, there is not one commonly accepted, standardized tool for rating the quality of studies. So, in the research papers, reviewers looked for an assessment of the quality of each study and a clear description of the process used.

**Question 6. List and describe included studies**

All included studies were listed in the review, along with descriptions of their key characteristics. This was presented either in narrative or table format.

**Question 7. Publication bias**

Publication bias is a term used when studies with positive results have a higher likelihood of being published, being published rapidly, being published in higher impact journals, being published in English, being published more than once, or being cited by others. Publication bias can be linked to favorable or unfavorable treatment of research findings due to investigators, editors, industry, commercial interests, or peer reviewers. To minimize the potential for publication bias, researchers can conduct a comprehensive literature search that includes the strategies discussed in Question 3.

A funnel plot–a scatter plot of component studies in a meta-analysis–is a commonly used graphical method for detecting publication bias. If there is no significant publication bias, the graph looks like a symmetrical inverted funnel.

Reviewers assessed and clearly described the likelihood of publication bias.

**Question 8. Heterogeneity**

Heterogeneity is used to describe important differences in studies included in a meta-analysis that may make it inappropriate to combine the studies. Heterogeneity can be clinical (e.g., important differences between study participants, baseline disease severity, and interventions); methodological (e.g., important differences in the design and conduct of the study); or statistical (e.g., important differences in the quantitative results or reported effects).

Researchers usually assess clinical or methodological heterogeneity qualitatively by determining whether it makes sense to combine studies. For example:

- Should a study evaluating the effects of an intervention on CVD risk that involves elderly male smokers with hypertension be combined with a study that involves healthy adults ages 18 to 40? (Clinical Heterogeneity)
- Should a study that uses a randomized controlled trial (RCT) design be combined with a study that uses a case-control study design? (Methodological Heterogeneity)

Statistical heterogeneity describes the degree of variation in the effect estimates from a set of studies; it is assessed quantitatively. The two most common methods used to assess statistical heterogeneity are the Q test (also known as the X2 or chi-square test) or I2 test.

Reviewers examined studies to determine if an assessment for heterogeneity was conducted and clearly described. If the studies are found to be heterogeneous, the investigators should explore and explain the causes of the heterogeneity, and determine what influence, if any, the study differences had on overall study results.

# Quality Assessment Tool for Observational Cohort and Cross-Sectional Studies

- Study Quality Assessment Tools

| **Criteria** | **Yes** | **No** | **Other (CD, NR, NA)*** | |
| --- | --- | --- | --- | --- |
| 1. Was the research question or objective in this paper clearly stated? |  |  |  |  |
| 2. Was the study population clearly specified and defined? |  |  |  |  |
| 3. Was the participation rate of eligible persons at least 50%? |  |  |  |  |
| 4. Were all the subjects selected or recruited from the same or similar populations (including the same time period)? Were inclusion and exclusion criteria for being in the study prespecified and applied uniformly to all participants? |  |  |  |  |
| 5. Was a sample size justification, power description, or variance and effect estimates provided? |  |  |  |  |
| 6. For the analyses in this paper, were the exposure(s) of interest measured prior to the outcome(s) being measured? |  |  |  |  |
| 7. Was the timeframe sufficient so that one could reasonably expect to see an association between exposure and outcome if it existed? |  |  |  |  |
| 8. For exposures that can vary in amount or level, did the study examine different levels of the exposure as related to the outcome (e.g., categories of exposure, or exposure measured as continuous variable)? |  |  |  |  |
| 9. Were the exposure measures (independent variables) clearly defined, valid, reliable, and implemented consistently across all study participants? |  |  |  |  |
| 10. Was the exposure(s) assessed more than once over time? |  |  |  |  |
| 11. Were the outcome measures (dependent variables) clearly defined, valid, reliable, and implemented consistently across all study participants? |  |  |  |  |
| 12. Were the outcome assessors blinded to the exposure status of participants? |  |  |  |  |
| 13. Was loss to follow-up after baseline 20% or less? |  |  |  |  |
| 14. Were key potential confounding variables measured and adjusted statistically for their impact on the relationship between exposure(s) and outcome(s)? |  |  |  |  |

| **Quality Rating (Good, Fair, or Poor)** |
| --- |
| Rater #1 initials: |
| Rater #2 initials: |
| Additional Comments (If POOR, please state why): |

*CD, cannot determine; NA, not applicable; NR, not reported

**Guidance for Assessing the Quality of Observational Cohort and Cross-Sectional Studies**

The guidance document below is organized by question number from the tool for quality assessment of observational cohort and cross-sectional studies.

**Question 1. Research question**

Did the authors describe their goal in conducting this research? Is it easy to understand what they were looking to find? This issue is important for any scientific paper of any type. Higher quality scientific research explicitly defines a research question.

**Questions 2 and 3. Study population**

Did the authors describe the group of people from which the study participants were selected or recruited, using demographics, location, and time period? If you were to conduct this study again, would you know who to recruit, from where, and from what time period? Is the cohort population free of the outcomes of interest at the time they were recruited?

An example would be men over 40 years old with type 2 diabetes who began seeking medical care at Phoenix Good Samaritan Hospital between January 1, 1990 and December 31, 1994. In this example, the population is clearly described as: (1) who (men over 40 years old with type 2 diabetes); (2) where (Phoenix Good Samaritan Hospital); and (3) when (between January 1, 1990 and December 31, 1994). Another example is women ages 34 to 59 years of age in 1980 who were in the nursing profession and had no known coronary disease, stroke, cancer, hypercholesterolemia, or diabetes, and were recruited from the 11 most populous States, with contact information obtained from State nursing boards.

In cohort studies, it is crucial that the population at baseline is free of the outcome of interest. For example, the nurses' population above would be an appropriate group in which to study incident coronary disease. This information is usually found either in descriptions of population recruitment, definitions of variables, or inclusion/exclusion criteria.

You may need to look at prior papers on methods in order to make the assessment for this question. Those papers are usually in the reference list.

If fewer than 50% of eligible persons participated in the study, then there is concern that the study population does not adequately represent the target population. This increases the risk of bias.

**Question 4. Groups recruited from the same population and uniform eligibility criteria**

Were the inclusion and exclusion criteria developed prior to recruitment or selection of the study population? Were the same underlying criteria used for all of the subjects involved? This issue is related to the description of the study population, above, and you may find the information for both of these questions in the same section of the paper.

Most cohort studies begin with the selection of the cohort; participants in this cohort are then measured or evaluated to determine their exposure status. However, some cohort studies may recruit or select exposed participants in a different time or place than unexposed participants, especially retrospective cohort studies–which is when data are obtained from the past (retrospectively), but the analysis examines exposures prior to outcomes. For example, one research question could be whether diabetic men with clinical depression are at higher risk for cardiovascular disease than those without clinical depression. So, diabetic men with depression might be selected from a mental health clinic, while diabetic men without depression might be selected from an internal medicine or endocrinology clinic. This study recruits groups from different clinic populations, so this example would get a "no."

However, the women nurses described in the question above were selected based on the same inclusion/exclusion criteria, so that example would get a "yes."

**Question 5. Sample size justification**

Did the authors present their reasons for selecting or recruiting the number of people included or analyzed? Do they note or discuss the statistical power of the study? This question is about whether or not the study had enough participants to detect an association if one truly existed.

A paragraph in the methods section of the article may explain the sample size needed to detect a hypothesized difference in outcomes. You may also find a discussion of power in the discussion section (such as the study had 85% power to detect a 20% increase in the rate of an outcome of interest, with a 2-sided alpha of 0.05). Sometimes estimates of variance and/or estimates of effect size are given, instead of sample size calculations. In any of these cases, the answer would be "yes."

However, observational cohort studies often do not report anything about power or sample sizes because the analyses are exploratory in nature. In this case, the answer would be "no." This is not a "fatal flaw." It just may indicate that attention was not paid to whether the study was sufficiently sized to answer a prespecified question–i.e., it may have been an exploratory, hypothesis-generating study.

**Question 6. Exposure assessed prior to outcome measurement**

This question is important because, in order to determine whether an exposure causes an outcome, the exposure must come before the outcome.

For some prospective cohort studies, the investigator enrolls the cohort and then determines the exposure status of various members of the cohort (large epidemiological studies like Framingham used this approach). However, for other cohort studies, the cohort is selected based on its exposure status, as in the example above of depressed diabetic men (the exposure being depression). Other examples include a cohort identified by its exposure to fluoridated drinking water and then compared to a cohort living in an area without fluoridated water, or a cohort of military personnel exposed to combat in the Gulf War compared to a cohort of military personnel not deployed in a combat zone.

With either of these types of cohort studies, the cohort is followed forward in time (i.e., prospectively) to assess the outcomes that occurred in the exposed members compared to nonexposed members of the cohort. Therefore, you begin the study in the present by looking at groups that were exposed (or not) to some biological or behavioral factor, intervention, etc., and then you follow them forward in time to examine outcomes. If a cohort study is conducted properly, the answer to this question should be "yes," since the exposure status of members of the cohort was determined at the beginning of the study before the outcomes occurred.

For retrospective cohort studies, the same principal applies. The difference is that, rather than identifying a cohort in the present and following them forward in time, the investigators go back in time (i.e., retrospectively) and select a cohort based on their exposure status in the past and then follow them forward to assess the outcomes that occurred in the exposed and nonexposed cohort members. Because in retrospective cohort studies the exposure and outcomes may have already occurred (it depends on how long they follow the cohort), it is important to make sure that the exposure preceded the outcome.

Sometimes cross-sectional studies are conducted (or cross-sectional analyses of cohort-study data), where the exposures and outcomes are measured during the same timeframe. As a result, cross-sectional analyses provide weaker evidence than regular cohort studies regarding a potential causal relationship between exposures and outcomes. For cross-sectional analyses, the answer to Question 6 should be "no."

**Question 7. Sufficient timeframe to see an effect**

Did the study allow enough time for a sufficient number of outcomes to occur or be observed, or enough time for an exposure to have a biological effect on an outcome? In the examples given above, if clinical depression has a biological effect on increasing risk for CVD, such an effect may take years. In the other example, if higher dietary sodium increases BP, a short timeframe may be sufficient to assess its association with BP, but a longer timeframe would be needed to examine its association with heart attacks.

The issue of timeframe is important to enable meaningful analysis of the relationships between exposures and outcomes to be conducted. This often requires at least several years, especially when looking at health outcomes, but it depends on the research question and outcomes being examined.

Cross-sectional analyses allow no time to see an effect, since the exposures and outcomes are assessed at the same time, so those would get a "no" response.

**Question 8. Different levels of the exposure of interest**

If the exposure can be defined as a range (examples: drug dosage, amount of physical activity, amount of sodium consumed), were multiple categories of that exposure assessed? (for example, for drugs: not on the medication, on a low dose, medium dose, high dose; for dietary sodium, higher than average U.S. consumption, lower than recommended consumption, between the two). Sometimes discrete categories of exposure are not used, but instead exposures are measured as continuous variables (for example, mg/day of dietary sodium or BP values).

In any case, studying different levels of exposure (where possible) enables investigators to assess trends or dose-response relationships between exposures and outcomes–e.g., the higher the exposure, the greater the rate of the health outcome. The presence of trends or dose-response relationships lends credibility to the hypothesis of causality between exposure and outcome.

For some exposures, however, this question may not be applicable (e.g., the exposure may be a dichotomous variable like living in a rural setting versus an urban setting, or vaccinated/not vaccinated with a one-time vaccine). If there are only two possible exposures (yes/no), then this question should be given an "NA," and it should not count negatively towards the quality rating.

**Question 9. Exposure measures and assessment**

Were the exposure measures defined in detail? Were the tools or methods used to measure exposure accurate and reliable–for example, have they been validated or are they objective? This issue is important as it influences confidence in the reported exposures. When exposures are measured with less accuracy or validity, it is harder to see an association between exposure and outcome even if one exists. Also as important is whether the exposures were assessed in the same manner within groups and between groups; if not, bias may result.

For example, retrospective self-report of dietary salt intake is not as valid and reliable as prospectively using a standardized dietary log plus testing participants' urine for sodium content. Another example is measurement of BP, where there may be quite a difference between usual care, where clinicians measure BP however it is done in their practice setting (which can vary considerably), and use of trained BP assessors using standardized equipment (e.g., the same BP device which has been tested and calibrated) and a standardized protocol (e.g., patient is seated for 5 minutes with feet flat on the floor, BP is taken twice in each arm, and all four measurements are averaged). In each of these cases, the former would get a "no" and the latter a "yes."

Here is a final example that illustrates the point about why it is important to assess exposures consistently across all groups: If people with higher BP (exposed cohort) are seen by their providers more frequently than those without elevated BP (nonexposed group), it also increases the chances of detecting and documenting changes in health outcomes, including CVD-related events. Therefore, it may lead to the conclusion that higher BP leads to more CVD events. This may be true, but it could also be due to the fact that the subjects with higher BP were seen more often; thus, more CVD-related events were detected and documented simply because they had more encounters with the health care system. Thus, it could bias the results and lead to an erroneous conclusion.

**Question 10. Repeated exposure assessment**

Was the exposure for each person measured more than once during the course of the study period? Multiple measurements with the same result increase our confidence that the exposure status was correctly classified. Also, multiple measurements enable investigators to look at changes in exposure over time, for example, people who ate high dietary sodium throughout the follow-up period, compared to those who started out high then reduced their intake, compared to those who ate low sodium throughout. Once again, this may not be applicable in all cases. In many older studies, exposure was measured only at baseline. However, multiple exposure measurements do result in a stronger study design.

**Question 11. Outcome measures**

Were the outcomes defined in detail? Were the tools or methods for measuring outcomes accurate and reliable–for example, have they been validated or are they objective? This issue is important because it influences confidence in the validity of study results. Also important is whether the outcomes were assessed in the same manner within groups and between groups.

An example of an outcome measure that is objective, accurate, and reliable is death–the outcome measured with more accuracy than any other. But even with a measure as objective as death, there can be differences in the accuracy and reliability of how death was assessed by the investigators. Did they base it on an autopsy report, death certificate, death registry, or report from a family member? Another example is a study of whether dietary fat intake is related to blood cholesterol level (cholesterol level being the outcome), and the cholesterol level is measured from fasting blood samples that are all sent to the same laboratory. These examples would get a "yes." An example of a "no" would be self-report by subjects that they had a heart attack, or self-report of how much they weigh (if body weight is the outcome of interest).

Similar to the example in Question 9, results may be biased if one group (e.g., people with high BP) is seen more frequently than another group (people with normal BP) because more frequent encounters with the health care system increases the chances of outcomes being detected and documented.

**Question 12. Blinding of outcome assessors**

Blinding means that outcome assessors did not know whether the participant was exposed or unexposed. It is also sometimes called "masking." The objective is to look for evidence in the article that the person(s) assessing the outcome(s) for the study (for example, examining medical records to determine the outcomes that occurred in the exposed and comparison groups) is masked to the exposure status of the participant. Sometimes the person measuring the exposure is the same person conducting the outcome assessment. In this case, the outcome assessor would most likely not be blinded to exposure status because they also took measurements of exposures. If so, make a note of that in the comments section.

As you assess this criterion, think about whether it is likely that the person(s) doing the outcome assessment would know (or be able to figure out) the exposure status of the study participants. If the answer is no, then blinding is adequate. An example of adequate blinding of the outcome assessors is to create a separate committee, whose members were not involved in the care of the patient and had no information about the study participants' exposure status. The committee would then be provided with copies of participants' medical records, which had been stripped of any potential exposure information or personally identifiable information. The committee would then review the records for prespecified outcomes according to the study protocol. If blinding was not possible, which is sometimes the case, mark "NA" and explain the potential for bias.

**Question 13. Follow-up rate**

Higher overall follow-up rates are always better than lower follow-up rates, even though higher rates are expected in shorter studies, whereas lower overall follow-up rates are often seen in studies of longer duration. Usually, an acceptable overall follow-up rate is considered 80% or more of participants whose exposures were measured at baseline. However, this is just a general guideline. For example, a 6-month cohort study examining the relationship between dietary sodium intake and BP level may have over 90% follow-up, but a 20-year cohort study examining effects of sodium intake on stroke may have only a 65% follow-up rate.

**Question 14. Statistical analyses**

Were key potential confounding variables measured and adjusted for, such as by statistical adjustment for baseline differences? Logistic regression or other regression methods are often used to account for the influence of variables not of interest.

This is a key issue in cohort studies, because statistical analyses need to control for potential confounders, in contrast to an RCT, where the randomization process controls for potential confounders. All key factors that may be associated both with the exposure of interest and the outcome–that are not of interest to the research question–should be controlled for in the analyses.

For example, in a study of the relationship between cardiorespiratory fitness and CVD events (heart attacks and strokes), the study should control for age, BP, blood cholesterol, and body weight, because all of these factors are associated both with low fitness and with CVD events. Well-done cohort studies control for multiple potential confounders.

**Some general guidance for determining the overall quality rating of observational cohort and cross-sectional studies**

The questions on the form are designed to help you focus on the key concepts for evaluating the internal validity of a study. They are not intended to create a list that you simply tally up to arrive at a summary judgment of quality.

Internal validity for cohort studies is the extent to which the results reported in the study can truly be attributed to the exposure being evaluated and not to flaws in the design or conduct of the study–in other words, the ability of the study to draw associative conclusions about the effects of the exposures being studied on outcomes. Any such flaws can increase the risk of bias.

Critical appraisal involves considering the risk of potential for selection bias, information bias, measurement bias, or confounding (the mixture of exposures that one cannot tease out from each other). Examples of confounding include co-interventions, differences at baseline in patient characteristics, and other issues throughout the questions above. High risk of bias translates to a rating of poor quality. Low risk of bias translates to a rating of good quality. (Thus, the greater the risk of bias, the lower the quality rating of the study.)

In addition, the more attention in the study design to issues that can help determine whether there is a causal relationship between the exposure and outcome, the higher quality the study. These include exposures occurring prior to outcomes, evaluation of a dose-response gradient, accuracy of measurement of both exposure and outcome, sufficient timeframe to see an effect, and appropriate control for confounding–all concepts reflected in the tool.

Generally, when you evaluate a study, you will not see a "fatal flaw," but you will find some risk of bias. By focusing on the concepts underlying the questions in the quality assessment tool, you should ask yourself about the potential for bias in the study you are critically appraising. For any box where you check "no" you should ask, "What is the potential risk of bias resulting from this flaw in study design or execution?" That is, does this factor cause you to doubt the results that are reported in the study or doubt the ability of the study to accurately assess an association between exposure and outcome?

The best approach is to think about the questions in the tool and how each one tells you something about the potential for bias in a study. The more you familiarize yourself with the key concepts, the more comfortable you will be with critical appraisal. Examples of studies rated good, fair, and poor are useful, but each study must be assessed on its own based on the details that are reported and consideration of the concepts for minimizing bias.

# Quality Assessment of Case-Control Studies

- Study Quality Assessment Tools

| **Criteria** | **Yes** | **No** | **Other (CD, NR, NA)*** |
| --- | --- | --- | --- |
| 1. Was the research question or objective in this paper clearly stated and appropriate? |  |  |  |
| 2. Was the study population clearly specified and defined? |  |  |  |
| 3. Did the authors include a sample size justification? |  |  |  |
| 4. Were controls selected or recruited from the same or similar population that gave rise to the cases (including the same timeframe)? |  |  |  |
| 5. Were the definitions, inclusion and exclusion criteria, algorithms or processes used to identify or select cases and controls valid, reliable, and implemented consistently across all study participants? |  |  |  |
| 6. Were the cases clearly defined and differentiated from controls? |  |  |  |
| 7. If less than 100 percent of eligible cases and/or controls were selected for the study, were the cases and/or controls randomly selected from those eligible? |  |  |  |
| 8. Was there use of concurrent controls? |  |  |  |
| 9. Were the investigators able to confirm that the exposure/risk occurred prior to the development of the condition or event that defined a participant as a case? |  |  |  |
| 10. Were the measures of exposure/risk clearly defined, valid, reliable, and implemented consistently (including the same time period) across all study participants? |  |  |  |
| 11. Were the assessors of exposure/risk blinded to the case or control status of participants? |  |  |  |
| 12. Were key potential confounding variables measured and adjusted statistically in the analyses? If matching was used, did the investigators account for matching during study analysis? |  |  |  |

| **Quality Rating (Good, Fair, or Poor) (see guidance)** |
| --- |
| Rater #1 Initials: |
| Rater #2 Initials: |
| Additional Comments (If POOR, please state why): |

*CD, cannot determine; NA, not applicable; NR, not reported

**Guidance for Assessing the Quality of Case-Control Studies**

The guidance document below is organized by question number from the tool for quality assessment of case-control studies.

**Question 1. Research question**

Did the authors describe their goal in conducting this research? Is it easy to understand what they were looking to find? This issue is important for any scientific paper of any type. High quality scientific research explicitly defines a research question.

**Question 2. Study population**

Did the authors describe the group of individuals from which the cases and controls were selected or recruited, while using demographics, location, and time period? If the investigators conducted this study again, would they know exactly who to recruit, from where, and from what time period?

Investigators identify case-control study populations by location, time period, and inclusion criteria for cases (individuals with the disease, condition, or problem) and controls (individuals without the disease, condition, or problem). For example, the population for a study of lung cancer and chemical exposure would be all incident cases of lung cancer diagnosed in patients ages 35 to 79, from January 1, 2003 to December 31, 2008, living in Texas during that entire time period, as well as controls without lung cancer recruited from the same population during the same time period. The population is clearly described as: (1) who (men and women ages 35 to 79 with (cases) and without (controls) incident lung cancer); (2) where (living in Texas); and (3) when (between January 1, 2003 and December 31, 2008).

Other studies may use disease registries or data from cohort studies to identify cases. In these cases, the populations are individuals who live in the area covered by the disease registry or included in a cohort study (i.e., nested case-control or case-cohort). For example, a study of the relationship between vitamin D intake and myocardial infarction might use patients identified via the GRACE registry, a database of heart attack patients.

NHLBI staff encouraged reviewers to examine prior papers on methods (listed in the reference list) to make this assessment, if necessary.

**Question 3. Target population and case representation**

In order for a study to truly address the research question, the target population–the population from which the study population is drawn and to which study results are believed to apply–should be carefully defined. Some authors may compare characteristics of the study cases to characteristics of cases in the target population, either in text or in a table. When study cases are shown to be representative of cases in the appropriate target population, it increases the likelihood that the study was well-designed per the research question.

However, because these statistics are frequently difficult or impossible to measure, publications should not be penalized if case representation is not shown. For most papers, the response to question 3 will be "NR." Those subquestions are combined because the answer to the second subquestion–case representation–determines the response to this item. However, it cannot be determined without considering the response to the first subquestion. For example, if the answer to the first subquestion is "yes," and the second, "CD," then the response for item 3 is "CD."

**Question 4. Sample size justification**

Did the authors discuss their reasons for selecting or recruiting the number of individuals included? Did they discuss the statistical power of the study and provide a sample size calculation to ensure that the study is adequately powered to detect an association (if one exists)? This question does not refer to a description of the manner in which different groups were included or excluded using the inclusion/exclusion criteria (e.g., "Final study size was 1,378 participants after exclusion of 461 patients with missing data" is not considered a sample size justification for the purposes of this question).

An article's methods section usually contains information on sample size and the size needed to detect differences in exposures and on statistical power.

**Question 5. Groups recruited from the same population**

To determine whether cases and controls were recruited from the same population, one can ask hypothetically, "If a control was to develop the outcome of interest (the condition that was used to select cases), would that person have been eligible to become a case?" Case-control studies begin with the selection of the cases (those with the outcome of interest, e.g., lung cancer) and controls (those in whom the outcome is absent). Cases and controls are then evaluated and categorized by their exposure status. For the lung cancer example, cases and controls were recruited from hospitals in a given region. One may reasonably assume that controls in the catchment area for the hospitals, or those already in the hospitals for a different reason, would attend those hospitals if they became a case; therefore, the controls are drawn from the same population as the cases. If the controls were recruited or selected from a different region (e.g., a State other than Texas) or time period (e.g., 1991-2000), then the cases and controls were recruited from different populations, and the answer to this question would be "no."

The following example further explores selection of controls. In a study, eligible cases were men and women, ages 18 to 39, who were diagnosed with atherosclerosis at hospitals in Perth, Australia, between July 1, 2000 and December 31, 2007. Appropriate controls for these cases might be sampled using voter registration information for men and women ages 18 to 39, living in Perth (population-based controls); they also could be sampled from patients without atherosclerosis at the same hospitals (hospital-based controls). As long as the controls are individuals who would have been eligible to be included in the study as cases (if they had been diagnosed with atherosclerosis), then the controls were selected appropriately from the same source population as cases.

In a prospective case-control study, investigators may enroll individuals as cases at the time they are found to have the outcome of interest; the number of cases usually increases as time progresses. At this same time, they may recruit or select controls from the population without the outcome of interest. One way to identify or recruit cases is through a surveillance system. In turn, investigators can select controls from the population covered by that system. This is an example of population-based controls. Investigators also may identify and select cases from a cohort study population and identify controls from outcome-free individuals in the same cohort study. This is known as a nested case-control study.

**Question 6. Inclusion and exclusion criteria prespecified and applied uniformly**

Were the inclusion and exclusion criteria developed prior to recruitment or selection of the study population? Were the same underlying criteria used for all of the groups involved? To answer this question, reviewers determined if the investigators developed I/E criteria prior to recruitment or selection of the study population and if they used the same underlying criteria for all groups. The investigators should have used the same selection criteria, except for study participants who had the disease or condition, which would be different for cases and controls by definition. Therefore, the investigators use the same age (or age range), gender, race, and other characteristics to select cases and controls. Information on this topic is usually found in a paper's section on the description of the study population.

**Question 7. Case and control definitions**

For this question, reviewers looked for descriptions of the validity of case and control definitions and processes or tools used to identify study participants as such. Was a specific description of "case" and "control" provided? Is there a discussion of the validity of the case and control definitions and the processes or tools used to identify study participants as such? They determined if the tools or methods were accurate, reliable, and objective. For example, cases might be identified as "adult patients admitted to a VA hospital from January 1, 2000 to December 31, 2009, with an ICD-9 discharge diagnosis code of acute myocardial infarction and at least one of the two confirmatory findings in their medical records: at least 2mm of ST elevation changes in two or more ECG leads and an elevated troponin level. Investigators might also use ICD-9 or CPT codes to identify patients. All cases should be identified using the same methods. Unless the distinction between cases and controls is accurate and reliable, investigators cannot use study results to draw valid conclusions.

**Question 8. Random selection of study participants**

If a case-control study did not use 100% of eligible cases and/or controls (e.g., not all disease-free participants were included as controls), did the authors indicate that random sampling was used to select controls? When it is possible to identify the source population fairly explicitly (e.g., in a nested case-control study, or in a registry-based study), then random sampling of controls is preferred. When investigators used consecutive sampling, which is frequently done for cases in prospective studies, then study participants are not considered randomly selected. In this case, the reviewers would answer "no" to Question 8. However, this would not be considered a fatal flaw.

If investigators included all eligible cases and controls as study participants, then reviewers marked "NA" in the tool. If 100% of cases were included (e.g., NA for cases) but only 50% of eligible controls, then the response would be "yes" if the controls were randomly selected, and "no" if they were not. If this cannot be determined, the appropriate response is "CD."

**Question 9. Concurrent controls**

A concurrent control is a control selected at the time another person became a case, usually on the same day. This means that one or more controls are recruited or selected from the population without the outcome of interest at the time a case is diagnosed. Investigators can use this method in both prospective case-control studies and retrospective case-control studies. For example, in a retrospective study of adenocarcinoma of the colon using data from hospital records, if hospital records indicate that Person A was diagnosed with adenocarcinoma of the colon on June 22, 2002, then investigators would select one or more controls from the population of patients without adenocarcinoma of the colon on that same day. This assumes they conducted the study retrospectively, using data from hospital records. The investigators could have also conducted this study using patient records from a cohort study, in which case it would be a nested case-control study.

Investigators can use concurrent controls in the presence or absence of matching and vice versa. A study that uses matching does not necessarily mean that concurrent controls were used.

**Question 10. Exposure assessed prior to outcome measurement**

Investigators first determine case or control status (based on presence or absence of outcome of interest), and then assess exposure history of the case or control; therefore, reviewers ascertained that the exposure preceded the outcome. For example, if the investigators used tissue samples to determine exposure, did they collect them from patients prior to their diagnosis? If hospital records were used, did investigators verify that the date a patient was exposed (e.g., received medication for atherosclerosis) occurred prior to the date they became a case (e.g., was diagnosed with type 2 diabetes)? For an association between an exposure and an outcome to be considered causal, the exposure must have occurred prior to the outcome.

**Question 11. Exposure measures and assessment**

Were the exposure measures defined in detail? Were the tools or methods used to measure exposure accurate and reliable–for example, have they been validated or are they objective? This is important, as it influences confidence in the reported exposures. Equally important is whether the exposures were assessed in the same manner within groups and between groups. This question pertains to bias resulting from exposure misclassification (i.e., exposure ascertainment).

For example, a retrospective self-report of dietary salt intake is not as valid and reliable as prospectively using a standardized dietary log plus testing participants' urine for sodium content because participants' retrospective recall of dietary salt intake may be inaccurate and result in misclassification of exposure status. Similarly, BP results from practices that use an established protocol for measuring BP would be considered more valid and reliable than results from practices that did not use standard protocols. A protocol may include using trained BP assessors, standardized equipment (e.g., the same BP device which has been tested and calibrated), and a standardized procedure (e.g., patient is seated for 5 minutes with feet flat on the floor, BP is taken twice in each arm, and all four measurements are averaged).

**Question 12. Blinding of exposure assessors**

Blinding or masking means that outcome assessors did not know whether participants were exposed or unexposed. To answer this question, reviewers examined articles for evidence that the outcome assessor(s) was masked to the exposure status of the research participants. An outcome assessor, for example, may examine medical records to determine the outcomes that occurred in the exposed and comparison groups. Sometimes the person measuring the exposure is the same person conducting the outcome assessment. In this case, the outcome assessor would most likely not be blinded to exposure status. A reviewer would note such a finding in the comments section of the assessment tool.

One way to ensure good blinding of exposure assessment is to have a separate committee, whose members have no information about the study participants' status as cases or controls, review research participants' records. To help answer the question above, reviewers determined if it was likely that the outcome assessor knew whether the study participant was a case or control. If it was unlikely, then the reviewers marked "no" to Question 12. Outcome assessors who used medical records to assess exposure should not have been directly involved in the study participants' care, since they probably would have known about their patients' conditions. If the medical records contained information on the patient's condition that identified him/her as a case (which is likely), that information would have had to be removed before the exposure assessors reviewed the records.

If blinding was not possible, which sometimes happens, the reviewers marked "NA" in the assessment tool and explained the potential for bias.

**Question 13. Statistical analysis**

Were key potential confounding variables measured and adjusted for, such as by statistical adjustment for baseline differences? Investigators often use logistic regression or other regression methods to account for the influence of variables not of interest.

This is a key issue in case-controlled studies; statistical analyses need to control for potential confounders, in contrast to RCTs in which the randomization process controls for potential confounders. In the analysis, investigators need to control for all key factors that may be associated with both the exposure of interest and the outcome and are not of interest to the research question.

A study of the relationship between smoking and CVD events illustrates this point. Such a study needs to control for age, gender, and body weight; all are associated with smoking and CVD events. Well-done case-control studies control for multiple potential confounders.

Matching is a technique used to improve study efficiency and control for known confounders. For example, in the study of smoking and CVD events, an investigator might identify cases that have had a heart attack or stroke and then select controls of similar age, gender, and body weight to the cases. For case-control studies, it is important that if matching was performed during the selection or recruitment process, the variables used as matching criteria (e.g., age, gender, race) should be controlled for in the analysis.

**General Guidance for Determining the Overall Quality Rating of Case-Controlled Studies**

NHLBI designed the questions in the assessment tool to help reviewers focus on the key concepts for evaluating a study's internal validity, not to use as a list from which to add up items to judge a study's quality.

Internal validity for case-control studies is the extent to which the associations between disease and exposure reported in the study can truly be attributed to the exposure being evaluated rather than to flaws in the design or conduct of the study. In other words, what is ability of the study to draw associative conclusions about the effects of the exposures on outcomes? Any such flaws can increase the risk of bias.

In critical appraising a study, the following factors need to be considered: risk of potential for selection bias, information bias, measurement bias, or confounding (the mixture of exposures that one cannot tease out from each other). Examples of confounding include co-interventions, differences at baseline in patient characteristics, and other issues addressed in the questions above. High risk of bias translates to a poor quality rating; low risk of bias translates to a good quality rating. Again, the greater the risk of bias, the lower the quality rating of the study.

In addition, the more attention in the study design to issues that can help determine whether there is a causal relationship between the outcome and the exposure, the higher the quality of the study. These include exposures occurring prior to outcomes, evaluation of a dose-response gradient, accuracy of measurement of both exposure and outcome, sufficient timeframe to see an effect, and appropriate control for confounding–all concepts reflected in the tool.

If a study has a "fatal flaw," then risk of bias is significant; therefore, the study is deemed to be of poor quality. An example of a fatal flaw in case-control studies is a lack of a consistent standard process used to identify cases and controls.

Generally, when reviewers evaluated a study, they did not see a "fatal flaw," but instead found some risk of bias. By focusing on the concepts underlying the questions in the quality assessment tool, reviewers examined the potential for bias in the study. For any box checked "no," reviewers asked, "What is the potential risk of bias resulting from this flaw in study design or execution?" That is, did this factor lead to doubt about the results reported in the study or the ability of the study to accurately assess an association between exposure and outcome?

By examining questions in the assessment tool, reviewers were best able to assess the potential for bias in a study. Specific rules were not useful, as each study had specific nuances. In addition, being familiar with the key concepts helped reviewers assess the studies. Examples of studies rated good, fair, and poor were useful, yet each study had to be assessed on its own.

# Quality Assessment Tool for Before-After (Pre-Post) Studies With No Control Group

- Study Quality Assessment Tools

| **Criteria** | **Yes** | **No** | **Other (CD, NR, NA)*** |
| --- | --- | --- | --- |
| 1. Was the study question or objective clearly stated? |  |  |  |
| 2. Were eligibility/selection criteria for the study population prespecified and clearly described? |  |  |  |
| 3. Were the participants in the study representative of those who would be eligible for the test/service/intervention in the general or clinical population of interest? |  |  |  |
| 4. Were all eligible participants that met the prespecified entry criteria enrolled? |  |  |  |
| 5. Was the sample size sufficiently large to provide confidence in the findings? |  |  |  |
| 6. Was the test/service/intervention clearly described and delivered consistently across the study population? |  |  |  |
| 7. Were the outcome measures prespecified, clearly defined, valid, reliable, and assessed consistently across all study participants? |  |  |  |
| 8. Were the people assessing the outcomes blinded to the participants' exposures/interventions? |  |  |  |
| 9. Was the loss to follow-up after baseline 20% or less? Were those lost to follow-up accounted for in the analysis? |  |  |  |
| 10. Did the statistical methods examine changes in outcome measures from before to after the intervention? Were statistical tests done that provided p values for the pre-to-post changes? |  |  |  |
| 11. Were outcome measures of interest taken multiple times before the intervention and multiple times after the intervention (i.e., did they use an interrupted time-series design)? |  |  |  |
| 12. If the intervention was conducted at a group level (e.g., a whole hospital, a community, etc.) did the statistical analysis take into account the use of individual-level data to determine effects at the group level? |  |  |  |

| **Quality Rating (Good, Fair, or Poor) (see guidance)** |
| --- |
| Rater #1 Initials: |
| Rater #2 Initials: |
| Additional Comments (If POOR, please state why): |

*CD, cannot determine; NA, not applicable; NR, not reported

**Guidance for Assessing the Quality of Before-After (Pre-Post) Studies With No Control Group**

The guidance document below is organized by question number from the tool for quality assessment of controlled intervention studies.

**Question 1. Study question**

Did the authors describe their goal in conducting this research? Is it easy to understand what they were looking to find? This issue is important for any scientific paper of any type. Higher quality scientific research explicitly defines a research question.

**Question 2. Eligibility criteria and study population**

Did the authors describe the eligibility criteria applied to the individuals from whom the study participants were selected or recruited? In other words, if the investigators were to conduct this study again, would they know whom to recruit, from where, and from what time period?

Here is a sample description of a study population: men over age 40 with type 2 diabetes, who began seeking medical care at Phoenix Good Samaritan Hospital, between January 1, 2005 and December 31, 2007. The population is clearly described as: (1) who (men over age 40 with type 2 diabetes); (2) where (Phoenix Good Samaritan Hospital); and (3) when (between January 1, 2005 and December 31, 2007). Another sample description is women who were in the nursing profession, who were ages 34 to 59 in 1995, had no known CHD, stroke, cancer, hypercholesterolemia, or diabetes, and were recruited from the 11 most populous States, with contact information obtained from State nursing boards.

To assess this question, reviewers examined prior papers on study methods (listed in reference list) when necessary.

**Question 3. Study participants representative of clinical populations of interest**

The participants in the study should be generally representative of the population in which the intervention will be broadly applied. Studies on small demographic subgroups may raise concerns about how the intervention will affect broader populations of interest. For example, interventions that focus on very young or very old individuals may affect middle-aged adults differently. Similarly, researchers may not be able to extrapolate study results from patients with severe chronic diseases to healthy populations.

**Question 4. All eligible participants enrolled**

To further explore this question, reviewers may need to ask: Did the investigators develop the I/E criteria prior to recruiting or selecting study participants? Were the same underlying I/E criteria used for all research participants? Were all subjects who met the I/E criteria enrolled in the study?

**Question 5. Sample size**

Did the authors present their reasons for selecting or recruiting the number of individuals included or analyzed? Did they note or discuss the statistical power of the study? This question addresses whether there was a sufficient sample size to detect an association, if one did exist.

An article's methods section may provide information on the sample size needed to detect a hypothesized difference in outcomes and a discussion on statistical power (such as, the study had 85% power to detect a 20% increase in the rate of an outcome of interest, with a 2-sided alpha of 0.05). Sometimes estimates of variance and/or estimates of effect size are given, instead of sample size calculations. In any case, if the reviewers determined that the power was sufficient to detect the effects of interest, then they would answer "yes" to Question 5.

**Question 6. Intervention clearly described**

Another pertinent question regarding interventions is: Was the intervention clearly defined in detail in the study? Did the authors indicate that the intervention was consistently applied to the subjects? Did the research participants have a high level of adherence to the requirements of the intervention? For example, if the investigators assigned a group to 10 mg/day of Drug A, did most participants in this group take the specific dosage of Drug A? Or did a large percentage of participants end up not taking the specific dose of Drug A indicated in the study protocol?

Reviewers ascertained that changes in study outcomes could be attributed to study interventions. If participants received interventions that were not part of the study protocol and could affect the outcomes being assessed, the results could be biased.

**Question 7. Outcome measures clearly described, valid, and reliable**

Were the outcomes defined in detail? Were the tools or methods for measuring outcomes accurate and reliable–for example, have they been validated or are they objective? This question is important because the answer influences confidence in the validity of study results.

An example of an outcome measure that is objective, accurate, and reliable is death–the outcome measured with more accuracy than any other. But even with a measure as objective as death, differences can exist in the accuracy and reliability of how investigators assessed death. For example, did they base it on an autopsy report, death certificate, death registry, or report from a family member? Another example of a valid study is one whose objective is to determine if dietary fat intake affects blood cholesterol level (cholesterol level being the outcome) and in which the cholesterol level is measured from fasting blood samples that are all sent to the same laboratory. These examples would get a "yes."

An example of a "no" would be self-report by subjects that they had a heart attack, or self-report of how much they weight (if body weight is the outcome of interest).

**Question 8. Blinding of outcome assessors**

Blinding or masking means that the outcome assessors did not know whether the participants received the intervention or were exposed to the factor under study. To answer the question above, the reviewers examined articles for evidence that the person(s) assessing the outcome(s) was masked to the participants' intervention or exposure status. An outcome assessor, for example, may examine medical records to determine the outcomes that occurred in the exposed and comparison groups. Sometimes the person applying the intervention or measuring the exposure is the same person conducting the outcome assessment. In this case, the outcome assessor would not likely be blinded to the intervention or exposure status. A reviewer would note such a finding in the comments section of the assessment tool.

In assessing this criterion, the reviewers determined whether it was likely that the person(s) conducting the outcome assessment knew the exposure status of the study participants. If not, then blinding was adequate. An example of adequate blinding of the outcome assessors is to create a separate committee whose members were not involved in the care of the patient and had no information about the study participants' exposure status. Using a study protocol, committee members would review copies of participants' medical records, which would be stripped of any potential exposure information or personally identifiable information, for prespecified outcomes.

**Question 9. Follow-up rate**

Higher overall follow-up rates are always desirable to lower follow-up rates, although higher rates are expected in shorter studies, and lower overall follow-up rates are often seen in longer studies. Usually an acceptable overall follow-up rate is considered 80% or more of participants whose interventions or exposures were measured at baseline. However, this is a general guideline.

In accounting for those lost to follow-up, in the analysis, investigators may have imputed values of the outcome for those lost to follow-up or used other methods. For example, they may carry forward the baseline value or the last observed value of the outcome measure and use these as imputed values for the final outcome measure for research participants lost to follow-up.

**Question 10. Statistical analysis**

Were formal statistical tests used to assess the significance of the changes in the outcome measures between the before and after time periods? The reported study results should present values for statistical tests, such as p values, to document the statistical significance (or lack thereof) for the changes in the outcome measures found in the study.

**Question 11. Multiple outcome measures**

Were the outcome measures for each person measured more than once during the course of the before and after study periods? Multiple measurements with the same result increase confidence that the outcomes were accurately measured.

**Question 12. Group-level interventions and individual-level outcome efforts**

Group-level interventions are usually not relevant for clinical interventions such as bariatric surgery, in which the interventions are applied at the individual patient level. In those cases, the questions were coded as "NA" in the assessment tool.

**General Guidance for Determining the Overall Quality Rating of Before-After Studies**

The questions in the quality assessment tool were designed to help reviewers focus on the key concepts for evaluating the internal validity of a study. They are not intended to create a list from which to add up items to judge a study's quality.

Internal validity is the extent to which the outcome results reported in the study can truly be attributed to the intervention or exposure being evaluated, and not to biases, measurement errors, or other confounding factors that may result from flaws in the design or conduct of the study. In other words, what is the ability of the study to draw associative conclusions about the effects of the interventions or exposures on outcomes?

Critical appraisal of a study involves considering the risk of potential for selection bias, information bias, measurement bias, or confounding (the mixture of exposures that one cannot tease out from each other). Examples of confounding include co-interventions, differences at baseline in patient characteristics, and other issues throughout the questions above. High risk of bias translates to a rating of poor quality; low risk of bias translates to a rating of good quality. Again, the greater the risk of bias, the lower the quality rating of the study.

In addition, the more attention in the study design to issues that can help determine if there is a causal relationship between the exposure and outcome, the higher quality the study. These issues include exposures occurring prior to outcomes, evaluation of a dose-response gradient, accuracy of measurement of both exposure and outcome, and sufficient timeframe to see an effect.

Generally, when reviewers evaluate a study, they will not see a "fatal flaw," but instead will find some risk of bias. By focusing on the concepts underlying the questions in the quality assessment tool, reviewers should ask themselves about the potential for bias in the study they are critically appraising. For any box checked "no" reviewers should ask, "What is the potential risk of bias resulting from this flaw in study design or execution?" That is, does this factor lead to doubt about the results reported in the study or doubt about the ability of the study to accurately assess an association between the intervention or exposure and the outcome?

The best approach is to think about the questions in the assessment tool and how each one reveals something about the potential for bias in a study. Specific rules are not useful, as each study has specific nuances. In addition, being familiar with the key concepts will help reviewers be more comfortable with critical appraisal. Examples of studies rated good, fair, and poor are useful, but each study must be assessed on its own.

# Quality Assessment Tool for Case Series Studies

- Study Quality Assessment Tools

| **Criteria** | **Yes** | **No** | **Other (CD, NR, NA)*** |
| --- | --- | --- | --- |
| 1. Was the study question or objective clearly stated? |  |  |  |
| 2. Was the study population clearly and fully described, including a case definition? |  |  |  |
| 3. Were the cases consecutive? |  |  |  |
| 4. Were the subjects comparable? |  |  |  |
| 5. Was the intervention clearly described? |  |  |  |
| 6. Were the outcome measures clearly defined, valid, reliable, and implemented consistently across all study participants? |  |  |  |
| 7. Was the length of follow-up adequate? |  |  |  |
| 8. Were the statistical methods well-described? |  |  |  |
| 9. Were the results well-described? |  |  |  |

| **Quality Rating (Good, Fair, or Poor)** |
| --- |
| Rater #1 initials: |
| Rater #2 initials: |
| Additional Comments (If POOR, please state why): |

*CD, cannot determine; NA, not applicable; NR, not reported
